# Long-Term Exercise Mitigates Energy Expenditure and Inflammatory Responses Induced by Sleep Deprivation in Mice

**DOI:** 10.3390/biom15060862

**Published:** 2025-06-13

**Authors:** Tian-Shu Zheng, Xin-Ran Gao, Chen Gu, Yu-Ning Ru, Rui-Ping Xu, Yu-Hang Yang, De-Hua Wang

**Affiliations:** 1 School of Life Sciences, State Key Laboratory of Microbial Technology, Shandong University, No. 72 Binhai Road, Jimo District, Qingdao 266237, China; 202120357@mail.sdu.edu.cn (T.-S.Z.); 202212454@mail.sdu.edu.cn (X.-R.G.); 008172@yzu.edu.cn (C.G.); ruyuning@sdu.edu.cn (Y.-N.R.); 202320424@mail.sdu.edu.cn (R.-P.X.); yangyuha24@mails.tsinghua.edu.cn (Y.-H.Y.); 2 College of Bioscience and Biotechnology, Yangzhou University, Yangzhou 225000, China

**Keywords:** sleep deprivation, inflammation, moderate-intensity continuous training, gut microbiota, mice

## Abstract

**Background**: Sleep deprivation (SD), defined as the disruption or loss of normal sleep, negatively affects energy metabolism, immune function, and gut microbiota in both humans and animals. Although SD has detrimental effects, it is often unavoidable due to work or study demands. Exercise has been shown to improve sleep quality, regulate metabolism, and enhance immune function. However, whether exercise can mitigate the adverse effects of unavoidable SD remains unclear. **Methods**: To explore the protective effects of exercise against SD-induced gut microbiota and metabolic dysfunction, mice were randomly assigned to four groups: control (CTR), exercise (EXE), SD, and exercise + SD (EXE + SD). Inflammatory markers and gut microbiota composition were analyzed to assess the impacts of SD and exercise interventions. **Results**: The inflammatory levels and energy metabolism in SD mice were significantly increased compared to those in CTR mice. Compared with SD mice, EXE + SD mice had a more stable gut microbiota structure and higher butyrate levels. Meanwhile, the inflammatory response caused by SD was also inhibited by exercise preconditioning. Both lipopolysaccharide inhibitors injection and butyrate supplementation can partially alleviate the elevation of inflammatory response and energy metabolism caused by SD. **Conclusion**: The inflammation and energy metabolism disorders in mice caused by SD can be inhibited by exercise preconditioning through stabilizing the structure of gut microbiota. This protective effect is highly likely related to the increase in butyric acid levels caused by exercise.

## 1. Introduction

Sleep is a crucial physiological process for both humans and animals. With the demands of modern society, the pressure of work and study has intensified, leading to an increase in sleep-related issues, such as Alzheimer’s disease and depression [1,2,3]. Sleep deprivation (SD), defined as partial or total sleep loss, can arise from various factors, including environmental conditions, lifestyle choices, and psychological stress [4]. The consequences of SD are profound, as it impairs cognitive, immune, and cardiovascular functions [5,6,7]. However, SD remains unavoidable in many real-world scenarios. For example, students sacrifice sleep for exam preparation [8], and long-haul truck drivers or workers on night shifts face demanding tasks [9]. In these contexts, the inability to regain lost sleep can lead to severe impairments in physiological and behavioral functions [10]. Thus, there is an urgent need for effective solutions to counteract the negative effects of SD and its correlated health risks.

SD disrupts the circadian rhythm and leads to subsequent disturbances in thermoregulation, endocrine function, and energy metabolism [11,12], despite causing an increase in energy expenditure. It decreases the serum leptin level, significantly increasing hunger and appetite, contributing to metabolic disorders such as type 2 diabetes and obesity [13,14]. However, unlike in humans, research on mice reveals a contrasting finding that SD does not induce obesity in mice but instead significantly elevates their energy expenditure, resulting in weight loss [15,16], consistent with the role of sleep in energy conservation and tissue maintenance [17]. Increasing evidence suggests that SD-induced systemic inflammation plays a crucial role in disrupting the homeostasis of energy metabolism [18,19,20]. SD activates various inflammatory pathways, including TLR4/NF-κB and IL-1, leading to enhanced energy metabolism within immune cells, increased glycolysis, and decreased oxidative phosphorylation, which collectively facilitate rapid ATP and biosynthetic material production to support immune defense and tissue repair [21,22,23]. The activation of inflammatory pathways caused by SD not only reverses insulin signaling and metabolic pathways, leading to abnormal glucose and lipid metabolism, but also results in excessively high cytokine levels, eventually leading to death [24,25]. Additionally, SD alters the composition of the gut microbiota [26], resulting in reduced microbial diversity and abundance of beneficial bacteria [27], which further disrupts physiological processes [25,28]. Increased levels of lipopolysaccharides (LPS) and decreased concentrations of melatonin and short-chain fatty acids (SCFAs) have been noted following SD, leading to inflammation, cognitive impairment, and systemic dysregulation [29,30,31].

Despite the well-known adverse effects of SD, effective preventive strategies remain limited. Treatments such as melatonin and acetate supplementation, along with lifestyle modifications such as exercise, can mitigate some of the negative impacts of SD [32,33].

Exercise is recognized as a vital regulator of the body’s homeostasis, enhancing circulation and promoting the delivery of oxygen and nutrients while aiding in the clearance of metabolic waste and inflammatory mediators [34]. Numerous studies have demonstrated the beneficial effects of exercise on sleep quality, with the exception of high-intensity workouts [35]. Almost all forms of regular exercise, including short-term aerobic activities, resistance training, and mind–body exercises, show significant improvements in sleep quality, especially in reducing insomnia and sleep-disordered breathing [36]. Among various exercise intensities, moderate-intensity continuous training (MICT) is particularly beneficial for improving sleep quality and reducing occurrences of sleep disruptions and difficulty falling asleep [37,38]. Besides sleep problems, exercise also has a significant role in alleviating the various negative effects caused by SD, acting against various diseases, including metabolic disorders, cancer, and mood disturbances [26,39]. The metabolic health benefits of exercise are attributed to the combined actions of multiple systems, leading to comprehensive improvements in metabolic function and a reduced risk of chronic diseases [40]. Exercise has been shown to reduce inflammation by decreasing adipose tissue, which lowers the release of pro-inflammatory cytokines like TNF-α. Additionally, muscle contraction during exercise stimulates the release of anti-inflammatory cytokines, such as IL-1RA and IL-10 [41]. The levels of these cytokines remain elevated in the body, boosting the anti-inflammatory response. Exercise also increases kynurenic acid levels, which helps reduce inflammation caused by LPS [42]. Therefore, exercise presents a promising solution for preventing energy metabolism disorders and inflammatory responses resulting from SD. Emerging evidence shows that exercise not only benefits metabolism and immunity but also helps improve gut microbiota composition by increasing Bacteroidetes, lowering the Firmicutes/Bacteroidetes ratio, and reducing harmful bacteria such as *Blautia* [43]. It also enriches the diversity of the gut microbiota and enhances the abundance of beneficial bacteria, including *Prevotella*, *Methanobrevibacter*, and *Akkermansia* [44]. These changes in the gut microbiota induced by exercise can directly influence SCFA levels, increasing concentrations of butyrate and acetate [45,46], thereby regulating energy metabolism [47]. The elevation of SCFA levels induced by exercise can further enhance the anti-inflammatory capacity of the body and reduce related disease risks [48,49,50,51].

In our previous research, we observed that SD promotes lipolysis and enhances the thermogenic capacity of brown adipose tissue (BAT), along with a significant upregulation of energy metabolism [16]. We hypothesize that SD alters gut microbiota composition and increases systemic inflammation. This inflammatory response may, in turn, increase energy expenditure. Moreover, we postulate that long-term exercise training can mitigate the adverse effects of sleep deprivation by stabilizing gut microbiota and maintaining metabolite concentrations. Therefore, consistent exercise could alleviate discomfort and prevent related diseases in situations in which SD is unavoidable. Here, we conducted three experiments using C57BL/6J mice: SD treatment, exercise following SD, and SCFA supplementation during SD to evaluate inflammation levels, gut microbiota composition, and energy metabolism. We predict that MICT will enhance the stability of the gut microbiota, preserve beneficial bacteria during SD, reduce LPS production, alleviate inflammation, and maintain normal energy metabolism.

## 2. Materials and Methods

### 2.1. Animals

Six-week-old male C57BL/6J mice (Skbex Biotechnology Co., Ltd., Henan, China) were housed individually in plastic cages (35 × 20 × 15 cm^3^) under 12L:12D cycles (lights on from 8:00 to 20:00) at a constant ambient temperature of 23 ± 1 °C. The mice were anesthetized by inhalation with isoflurane and then euthanized. The animal procedures were approved by the Animal Ethics and Inspection Committee of Shandong University (SYDWLL-2021-96; approval date: 23 November 2021).

### 2.2. Animal Physical Exercise Training Protocol

All groups were acclimated to an eight-channel motor-driven treadmill (Sans A101C, Sions Biotechnology Co., Ltd., Jiangsu, China). We selected MICT as the primary intervention based on preliminary comparisons with other exercise modalities (Supplementary Figure S1 and Attached Tables S1–S3). The slope of the treadmill was set at 0%. The VO_2max_ of the mice was indirectly estimated by gradually increasing the treadmill speed until they could no longer maintain running, which was then used to determine the exercise intensity at different stages. Before initiating the formal MICT protocol, mice underwent a 3-day adaptive treadmill training phase. On the first day, mice ran at a speed of 6 m/min for 30 min; on the second day, at 6 m/min for 60 min; and on the third day, at 10 m/min for 30 min. Following adaptation, mice in the MICT group completed a structured 3-week training program, performed 5 days per week. Each session included a 4 min warm-up at 10 m/min (40–50% VO_2max_), a 50 min continuous running phase at 15 m/min (65–70% VO_2max_), and a 3 min cool-down at 6 m/min (30–40% VO_2max_). The total duration of each session was 57 min, covering approximately 808 m. The exercise treatment method was adapted from the training protocols described in previous studies [52,53,54], with modifications to ensure that the training velocity for MICT mice was maintained at 65–70% of their VO_2max_. To minimize the stress associated with treadmill exercise, only gentle tail touching was used to induce the mice to run, and no electric or voice stimulants were used. None of the animals were visibly hurt or died during the exercise session. The detailed exercise protocols are presented in tabular form in the supplementary (Attached Table S2).

### 2.3. Experimental Design and Sleep Deprivation Protocol

SD was performed according to our previous protocol [16]. In Experiment 1, twelve male mice with similar body weight were randomly assigned to the sleep-deprived group (SD, n = 6) and the control group (CTR, n = 6). The SD mice were housed in a sleep deprivation chamber (ZS-SM-II, Zhongshidichuang Science and Technology Development Co., Ltd., Beijing, China) with corn cob bedding in which a rotating bar is kept to deprive animals of total sleep. The bar was programmed to move at the speed of 7 r/min and to alternate between clockwise and counter-clockwise rotations for 7 days. After environmental adaptation, the mice remained stable, sleep deprivation was initiated at 9:00 a.m. and lasted for 7 days. The control mice were housed in a same-sized chamber but without the bar rotating. For each group, all mice were placed in a chamber for 7 days before the beginning of the experiment to allow for acclimation. Both groups were provided with food and water ad libitum during the experiment.

Experiment 2: Twenty-four 6-week-old male mice were randomly divided into four groups: the SD group (SD, n = 6), the non-treatment group (CTR, n = 6), the exercise group (EXE, n = 6), and the exercise plus sleep deprivation group (EXE + SD, n = 6). Mice in the EXE and EXE + SD groups were trained to run on a treadmill (Zhongshidichuang Science and Technology Development Co., Ltd., Beijing, China) following the MICT plan for 21 days. The EXE + SD and SD groups underwent the same sleep deprivation treatment as the SD group in Experiment 1. The CTR and EXE groups underwent the same treatment as the CTR groups in Experiment 1. The other mice were fed during the exercise training period. At the end of the final day of exercise training, the mice were returned to their home cages to resume normal food and water intake. After 3 h, the animals’ physiological state stabilized, after which body mass was measured and BMR was assessed. Sleep deprivation was initiated at 9:00 a.m. the following day. The post-experiment handling was the same as that in Experiment 1.

Experiment 3: Twenty-four 6-week-old male mice were randomly divided into four groups: the SD plus inhibitor group (SD + TAK 242, n = 6), the sleep deprivation plus butyrate gavage group (SD + Butyrate, n = 6), the sleep deprivation plus DMSO group (SD + DMSO, n = 6), and the sleep deprivation plus saline gavage group (SD + Saline, n = 6). The four groups underwent the same sleep deprivation treatment as in Experiment 1. During this period, the SD + TAK 242 group was administered an intraperitoneal injection on the first and fourth day (Merck, #614316, at a dose of 1 mg/kg). In contrast, the control group was administered a DMSO injection. The SD + Butyrate group was administered butyrate by gavage during the sleep deprivation period (Merck, # B5887, at a dose of 10 mg/kg). In contrast, the control group was administered saline by gavage. After the experiment, the procedures were the same as those used in Experiment 1. All groups were provided food (Keao Xieli Feed Co., Ltd., Tianjin, China) and water ad libitum during the experiment.

### 2.4. Body Weight and Food Intake

Both body weight and food intake were recorded between 9:00 and 10:00 every day during sleep deprivation treatment using an electronic balance (±0.1 g). The food residues from each group were collected and weighed after drying at 60 °C in the oven. Food intake was calculated as the amount of food fed the previous day minus the food residues and food left the next day.

### 2.5. Measurement of BMR

BMR was measured between 11:00 and 15:00 at the end of the experiments through oxygen consumption using an open-circuit respirometry system (TSE labmaster, Freistaat Thüringen, Germany) at 30 °C (within the thermal neutral zone of mice for 4 h). The airflow rate was 0.4 L/min. BMR was calculated by averaging a minimum of three consecutive, stable readings of oxygen consumption after 1 h of acclimation [16]. By analyzing the mice’s activity along the X, Y, and Z axes in the TSE system, we evaluated whether they exhibited signs of recovery sleep.

### 2.6. Tissue Collection and Body Fat Contents

Animals were sacrificed by asphyxiation with CO_2_ between 15:00 and 18:00 after being taken from the open-circuit respirometry system at the end of the experiment. The interscapular BAT (iBAT) and rectus femoris muscle were collected and immediately frozen using liquid nitrogen. Before inguinal WAT (iWAT), mesenteric WAT (mWAT), and epididymal WAT (eWAT) were collected, wet weights of the adipose tissues were taken with the hair removed (±1 mg). The remaining carcass, including the head and tail but excluding the reproductive organs, was weighed (±0.1 g).

### 2.7. Measurement of LPS, IL-6 Concentration in Serum

Blood was collected in 1.5 mL centrifugation tubes and placed at 4 °C overnight, followed by centrifugation at 3000 rpm for 30 min at 4 °C. The supernatant was collected and stored at −80 °C. According to the instructions, serum LPS and IL-6 concentrations were quantified using an ELISA kit (JL11317, Jianglai Biotechnology, Shanghai, China). Intra- and inter-assay coefficients of variation were both less than 10%.

### 2.8. Measurements of Gene Expression in BAT, Muscle by RT-qPCR

The total RNA of iBAT and muscle was extracted using TRIzol Reagent (G3013, Servicebio, Wuhan, China). After detecting RNA concentration, it was reverse-transcribed to cDNA by the SweScript All-in-One RT SuperMix for qPCR (G3337, Servicebio, Wuhan, China). RT-qPCR was performed using a LightCycle96 instrument (Roche, Basel, Switzerland) according to the manufacturer’s instructions. All samples were quantified for relative gene expression using GAPDH expression as an internal standard. The relative gene expression was determined using the 2^−ΔΔCt^ method.

### 2.9. Measurements of TLR4, NF-κB, IL-6, and GAPDH by Western Blot

Muscle proteins were extracted using radioimmunoprecipitation assay (RIPA) lysis buffer (G2002, Servicebio, Wuhan, China). Western blots of tissue lysates (20 μg protein of each sample) were incubated with primary antibodies (IL-6: 1:3000, ab233706, Abcam, Cambridge, MA, USA; NF-κB: 1:3000, ab32536, Abcam, Cambridge, MA, USA; TLR4: 1:3000, ab22048, Abcam, Cambridge, MA, USA; GAPDH: 1:5000, 60004-1-Ig, Proteintech, Wuhan, China) and incubated with the appropriate secondary antibodies (1:3000; E-AB-1003, E-AB-1008, Elabscience, Wuhan, China). ECL (34580, Thermo Fisher Scientific, Waltham, Massachusetts, USA) was used to detect chemiluminescence. Finally, Image J software v1.8.0 was used for gray value analysis, and GAPDH was used as an internal standard to calculate the relative expression of proteins.

### 2.10. Microbiota 16S rRNA Gene Sequencing

Microbial genomic DNA was isolated from cecum samples via the PowerSoil DNA Isolation Kit (#27100-4-EP, QIAGEN, Düsseldorf, Germany). The quantity and purity of DNA samples were determined using NanoDrop spectrophotometry (Thermo Fisher Scientific, Waltham, Massachusetts, USA). A High-Fidelity PCR system kit (#3553400001; Roche, Basel, Switzerland) was employed to amplify the 16S rRNA gene, using 15 ng of DNA as the template. The V3 and V4 hypervariable regions of the 16S rRNA gene were targeted with specific primers: the forward primer (5′-ACTCCTACGGGAGGCAGCAG-3′) and the reverse primer (5′-GGACTACHVGGGTWTCTAAT-3′). Post-amplification, the multiplexed PCR products were purified using the QIAquick Gel Extraction Kit (#28704, QIAGEN). Equimolar pooling of the purified amplicons was performed prior to paired-end sequencing on an Illumina MiSeq PE300 platform (Illumina, San Diego, USA). Sequencing data were processed by clustering sequences into operational taxonomic units (OTUs) at 97% sequence similarity using UPARSE 7.1. Taxonomic classification was performed across multiple ranks (phylum, class, order, family, genus, and species). To account for variability in sequencing depth, all samples were rarefied to 20,000 16S rRNA gene sequences prior to diversity analyses. Bioinformatic visualization and analysis were conducted using the Majorbio Cloud platform (https://cloud.majorbio.com, accessed on 10 March 2024). For Principal Coordinates Analysis (PCoA); data were standardized and converted into Euclidean distance matrices. Unsupervised Ward-linkage hierarchical clustering was applied to generate a heatmap using the R package “heatmap” version 1.0.12.

### 2.11. Determination of Intestinal SCFA Concentration

Cecal SCFAs (acetate, propionate, butyrate, iso-butyrate, valerate, and iso-valerate) were quantified by gas chromatography (Agilent 7890A, Agilent Technologies, Delaware, USA) equipped with an autosampler and flame ionization detection (FID) system. The analysis was performed using a capillary P-FFAP column (30 m × 0.25 mm × 0.25 µm, Agilent Technologies, Delaware, USA). Nitrogen (99.998%) was used as the carrier gas at a flow rate of 1.0 mL/min. The system was operated at an oven temperature of 250 °C. Injections were performed in splitless mode at 250 °C with a 0.5 µL injection volume. The oven temperature was programmed as follows: from 90 °C (1 min) at 10 °C/min to 120 °C (1 min), and then from 120 °C at 10 °C/min to 150 °C (3 min). SCFAs were identified by comparison with standard retention times, and their concentrations were quantified based on peak areas from calibration curves generated using known standards [55].

### 2.12. Statistical Analysis

IBM SPSS Statistics 19.0 and GraphPad Prism 8.0 were used for data statistical analysis. Variations in body weight were analyzed using repeated measures ANOVA. Carcass weight and BMR were analyzed by one-way ANCOVA with body mass as the covariate. Serum LPS and IL-6 levels, 16S rRNA sequence results, SCFAs concentration, gene expression, and protein levels were analyzed using independent two-sample Student’s *t*-test or one-way ANOVA. The results are presented as means ± SEM. A *p*-value of <0.05 was considered statistically significant.

## 3. Results

### 3.1. The Impact of SD on Energy Metabolism in Mice

**Figure 1:**
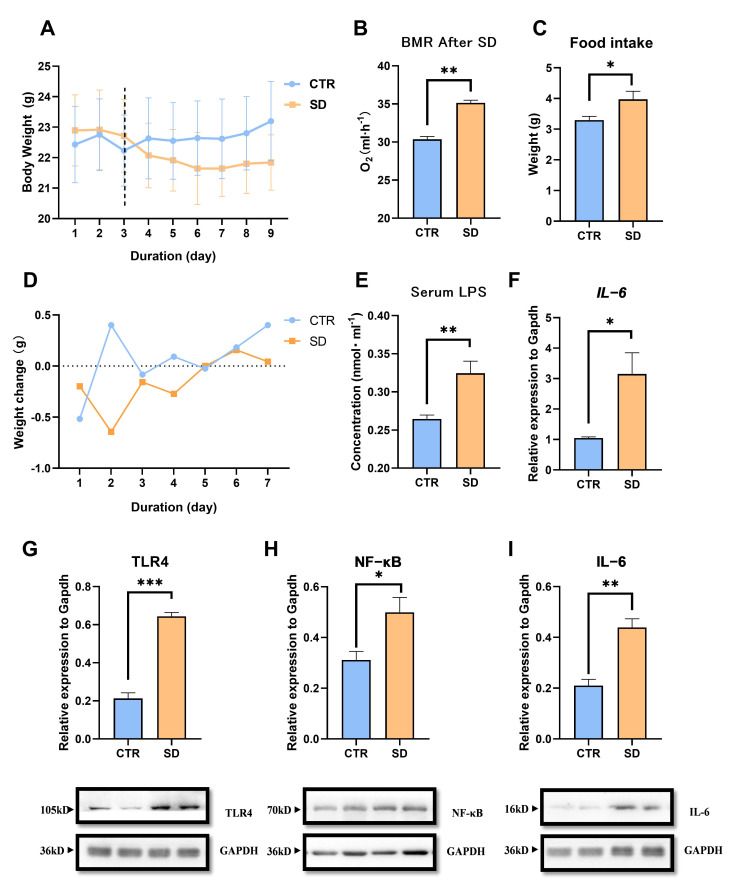
The effects of sleep deprivation on metabolism and inflammatory responses in mice: (**A**) body weight, (**B**) BMR after SD treatment, (**C**) daily average food intake, (**D**) body weight changes, (**E**) serum LPS level, (**F**) the gene expression level of IL-6 of muscle, (**G**–**I**) the protein levels of TLR4; NF-κB and IL-6 of muscle. Values are means ± SEM. *, *p* < 0.05, **, *p* < 0.01, ***, *p* < 0.001.

The body weight of the mice in the SD group was significantly lower than that of the CTR group (*t*_10_ = 2.485, *p* = 0.024, Figure 1A). BMR (*t*_10_ = 9.429, *p* < 0.0001, Figure 1B) and daily food intake (*t*_10_ = 3.582, *p* = 0.005, Figure 1C) were significantly higher in SD mice compared to the CTR group. Serum LPS levels (*t*_10_ = 3.582, *p* = 0.005, Figure 1E) were significantly higher in the SD group compared to the CTR group. SD increased the transcriptional levels of inflammatory factors in skeletal muscle, such as IL-6 (*t*_10_ = 3.023, *p* = 0.0128, Figure 1F). The results of protein levels indicated that SD activated the TLR4/NF-κB/IL-6 inflammatory pathway in skeletal muscle of mice (TLR4, *t*_10_ = 12.04, *p* < 0.0001, Figure 1G; NF-κB, *t*_10_ = 2.783, *p* = 0.0194, Figure 1H; IL-6, *t*_10_ = 5.416, *p* = 0.0003, Figure 1I).

### 3.2. The Inhibitory Effect of MICT on SD-Induced Detrimental Impacts

**Figure 2:**
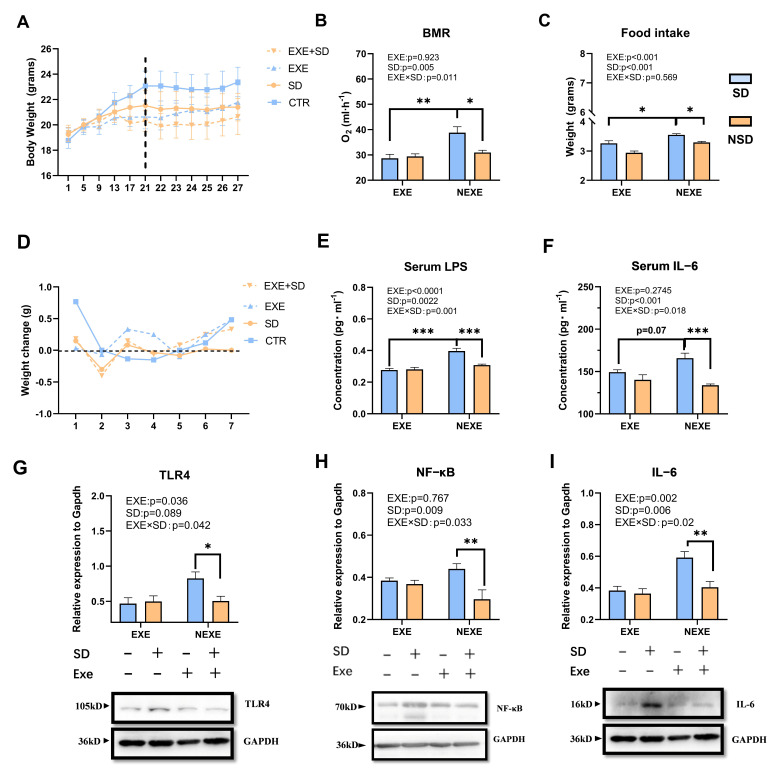
MICT prevented the impacts on inflammation and energy metabolism caused by SD: (**A**) body weight, (**B**) BMR, (**C**) daily average food intake, (**D**) body weight changes, (**E**) serum LPS level. (**F**) serumIL-6 level, (**G**–**I**) the protein levels of TLR4, NF-κB, and IL-6 of muscle. The labels above each column indicate the presence (+) or absence (−) of SD and Exe, respectively. Values are means ± SEM. *, *p* < 0.05, **, *p* < 0.01, ***, *p* < 0.001.

Compared with the other two exercise groups and the CTR group, 21-day MICT reduced the BMR of mice to a certain extent (*F*_(3,20)_ = 2.171, *p* = 0.0399, Figure S1A,B) and decreased serum LPS levels (*F*_(3,20)_ = 2.304, *p* = 0.044, Figure S1C). When MICT preconditioning is combined with SD, the body weight of SD group was significantly lower than in the CTR group (*F*_(3,20)_ = 9.295, *p* = 0.03, Figure 2A), while no significant difference was found between EXE + SD group and EXE group (*F*_(3,20)_ = 9.295, *p* = 0.556, Figure 2A). Compared to the SD group, the BMR in the EXE + SD group was significantly decreased (*F*_(3,20)_ = 7.975, *p* = 0.001, Figure 2B). Moreover, we observed an interaction between exercise and SD treatments (*p* = 0.011, Figure 2B). The daily food intake was significantly higher in the SD group compared to the EXE + SD group (*F*_(3,20)_ = 0.3355, *p* = 0.0144, Figure 2C). A significant interaction was found between exercise and SD in serum inflammatory markers (LPS, *p* = 0.001; IL-6, *p* = 0.018). The levels of LPS (*F*_(3,20)_ = 6.851, *p* =0.073, Figure 2E) and IL-6 (*F*_(3,20)_ = 14.88, *p* < 0.0001, Figure 2F) in the serum of the EXE + SD group were significantly lower compared to the SD group. The protein expression levels of TLR4 (*F*_(3,20)_ = 7.057, *p* = 0.0054, Figure 2G), NF-κB (*F*_(3,20)_ = 5.272, *p* = 0.0078, Figure 2H), and IL-6 (*F*_(3,20)_ = 4.846, *p* = 0.04, Figure 2I), which were reduced in the EXE + SD group.

### 3.3. The Modulation of MICT on the Gut Microbiota of SD Mice

**Figure 3:**
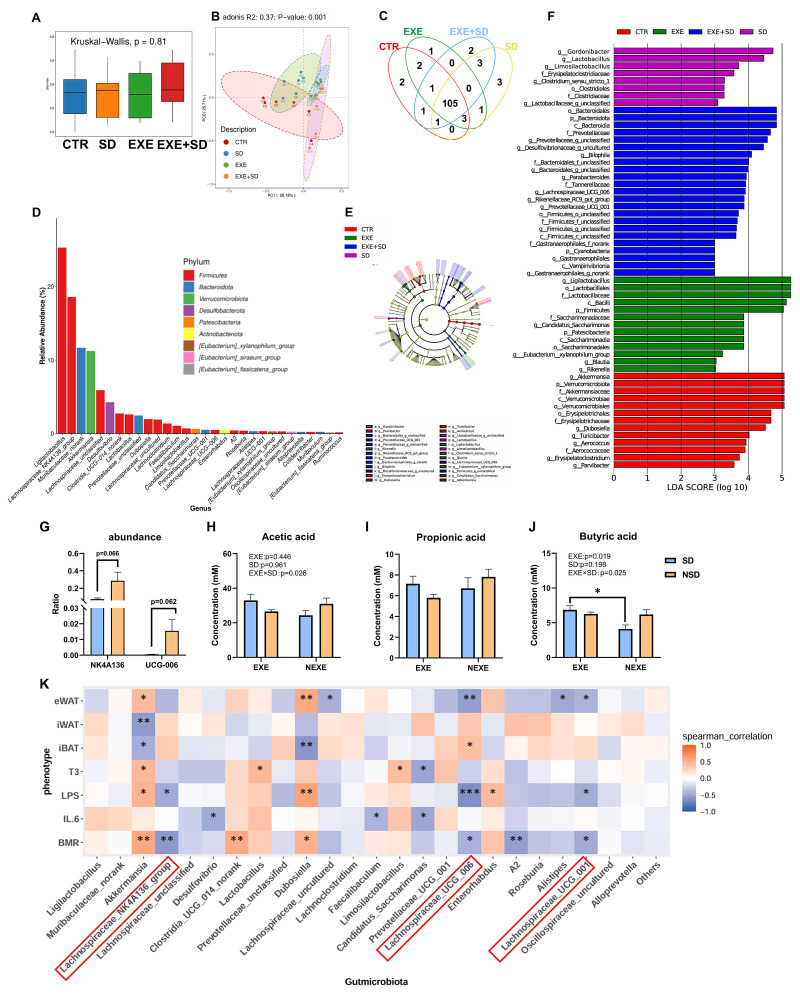
Influence of SD and MICT on the gut microbiota in mice. (**A**) Alpha diversity of bacterial communities across 4 groups based on Shannon score. (**B**) Principal coordinates analysis (PCoA) plots based on Bray–Curtis distance. (**C**) Differences in microbiota abundance of 4 groups. (**D**) Cladogram representing taxa enriched in the cecal microbiota community of the four groups detected by the LEfSe tool. (**E**) Relative abundance of the dominant genus. (**F**) Differential bacterial taxonomy was selected by LEfSe analysis with an LDA score of ≥3 in the cecal microbiota community of the four groups. (**G**) *Lachnospiraceae_NK4A136_group*, *Lachnospiraceae_UCG-006* abundance. (**H**–**J**) SCFAs level in gut. (**K**) Correlation analysis among phenotypes and microbiota abundance. The abundance of bacterial genus within the red-framed area showed a significant negative correlation with both LPS and BMR. Values are means ± SEM. *, *p* < 0.05, **, *p* < 0.01, ***, *p* < 0.001.

16S rRNA sequencing of the gut microbiota revealed significant differences in β-diversity among the four groups (*F*_(3,20)_ = 1.971, *p* = 0.001, Figure 3B), while no significant differences were found in α-diversity (*F*_(3,20)_ = 4.25, *p* = 0.81, Figure 3A). The Venn diagram showed an overlap between four groups; the presence of unique samples in each group further implies that SD and exercise may exert distinct influences on certain subsets of the sample population. (Figure 3C). Functional analysis using KEGG and COG pathways indicated that the differential microbiota in the groups was primarily enriched in pathways related to “Transcription”, “Carbohydrate transport and metabolism”, and “Signal transduction mechanisms” (Figure S4A). Phylogenetic tree showing the microbial community differences across four groups (Figure 3D). The relative abundance of the genera identified suggested that the microbial composition was heavily influenced by SD, which could explain the observed variations in the relative abundance of these genera (Figure 3E). SD group showed a lower abundance of beneficial bacteria compared to the EXE + SD groups (Figure 3F). Specifically, compared to the SD group, the abundances of *Lachnospiraceae_NK4A136_group* and *Lachnospiraceae_UCG-006* were significantly increased (*Lachnospiraceae_NK4A136_group*, *t*_10_ = 2.096, *p* = 0.0625; *Lachnospiraceae_UCG-006*, *t*_10_ = 2.058, *p* = 0.0667, Figure 3G). Additionally, the butyrate levels in the SD group were significantly decreased compared to the EXE + SD group (*t*_10_ = 2.67, *p* = 0.011, Figure 3J), indicating a potential role of butyrate in modulating the gut microbiota under SD conditions. The correlation analysis revealed a significant negative correlation between the abundance of several genera within the Lachnospiraceae family and both BMR and serum LPS levels (Figure 3K).

### 3.4. Butyrate Supplementation’s Inhibitory Effect of Metabolism and Inflammation on SD

**Figure 4:**
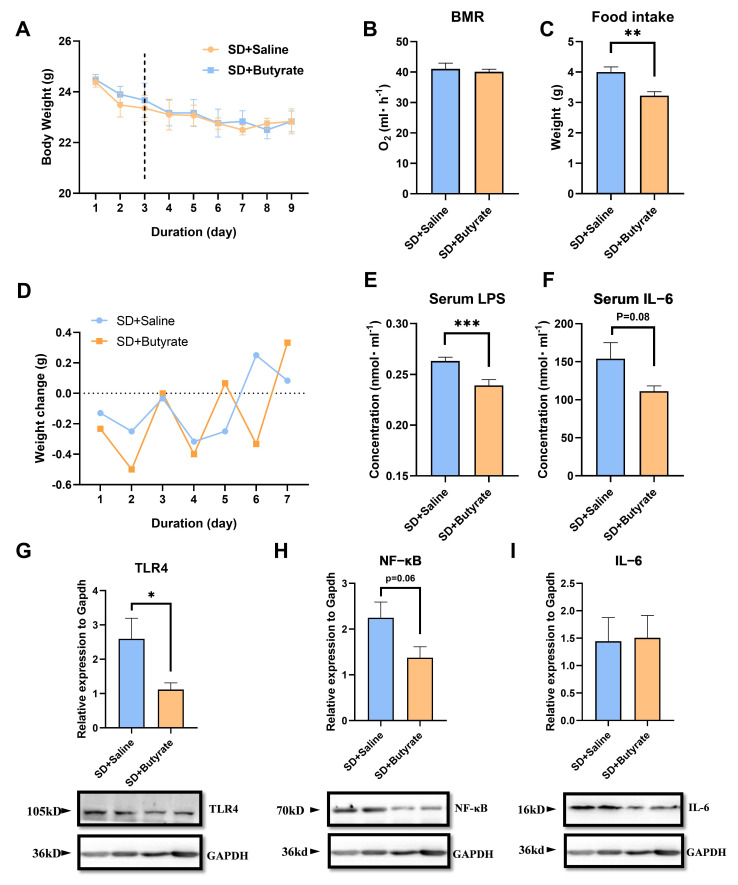
Effects of butyrate gavage on in SD mice: (**A**) body weight, (**B**) BMR,(**C**) daily average food intake, (**D**) body weight changes, (**E**) serum LPS level, (**F**) serum IL-6 level, (**G**–**I**) the protein levels ofTLR4; NF-κB and IL-6 of muscle. Values are means ± SEM. *, *p* < 0.05, **, *p* < 0.01, ***, *p* < 0.001.

Butyrate gavage treatment significantly reduced the increase in the average daily food intake caused by SD (*t*_10_ = 3.604, *p* < 0.01, Figure 4C). Although butyrate gavage did not alleviate the increase in BMR (*t*_10_ = 0.4605, *p* = 0.655, Figure 4B) and the decrease in body weight (*t*_10_ = 1.75, *p* = 0.1121, Figure 4A) caused by SD, it could effectively reduce the increase in LPS (*t*_10_ = 3.498, *p* = 0.005, Figure 4E) and IL-6 (*t*_10_ = 1.933, *p* = 0.082, Figure 4F) serum level caused by SD. Moreover, butyrate gavage treatment also inhibited the activation of the LPS downstream pathway in skeletal muscle (TLR4, *t*_10_ = 2.338, *p* = 0.0415, Figure 4G; NF-κB, *t*_10_ = 2.06, *p* = 0.0621, Figure 4H; IL-6, *t*_10_ = 0.2014, *p* = 0.8444, Figure 4I).

### 3.5. The Effects of Butyrate Supplementation on the Gut Microbiota of SD Mice

**Figure 5:**
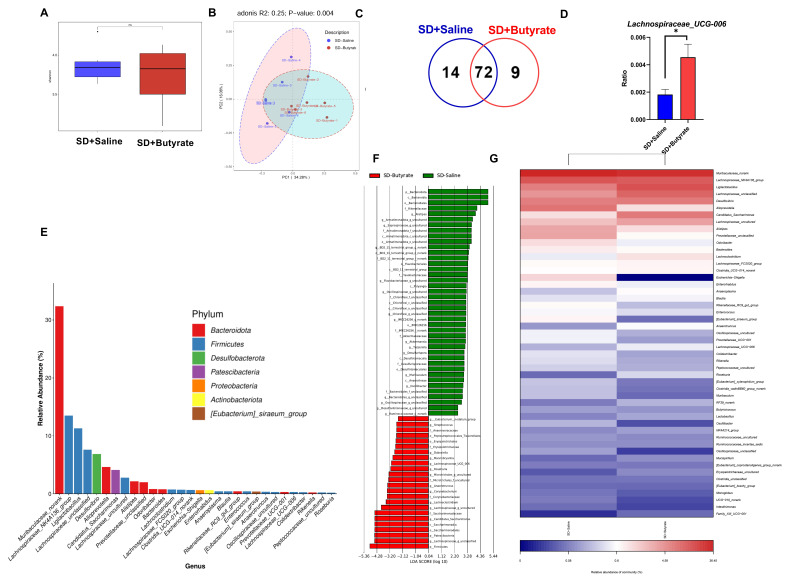
Influence of SD and butyrate gavage on gut microbiota in mice. (**A**) Alpha diversity of bacterial communities across two groups based on Shannon score. (**B**) Principal coordinates analysis (PCoA) plots based on Bray–Curtis distance. (**C**) Differences in microbiota abundance of 2 groups. (**D**) Difference in relative abundance of *Lachnospiraceae_UCG-006*. (**E**) Relative abundance of the dominant genus. (**F**) Differential bacterial taxonomy was selected by LEfSe analysis with an LDA score of ≥2.2 in the cecal microbiota community of the four groups. (**G**) Heatmap of top 50 different microbiota genera of 2 groups. Values are means ± SEM. *, *p* < 0.05.

The 16S rRNA results showed significant differences in the β-diversity of the intestinal microbiota between the Butyrate + SD and SD groups (*t*_10_ = 2.57, *p* = 0.001, Figure 5B), while no significant difference was found in α-diversity (*t*_10_ = 4.81, *p* > 0.05, Figure 5A). The Venn diagram showed the overlap between 2 groups (Figure 5C). The gavage of butyrate significantly reversed the decrease in the abundance of *Lachnospiraceae_UCG-006* caused by SD (*t*_10_ = 2.648, *p* = 0.024, Figure 5D). The relative abundance of the genera identified suggests that the microbial composition is significantly different between the two groups, which could explain the observed variations in the relative abundance of these genera (Figure 5E). The gavage of butyrate significantly increased the abundance of beneficial bacteria, including *Lactobacillus* and *Faecalibacterium* (Figure 5F). The SD + Saline group showed a lower abundance of beneficial bacteria such as *Lactobacillus* and *Lachnospiraceaee_UCG-006*, compared to the SD + Butyrate group (Figure 5G).

### 3.6. The Protective Effect of Inhibiting LPS in SD Mice

**Figure 6:**
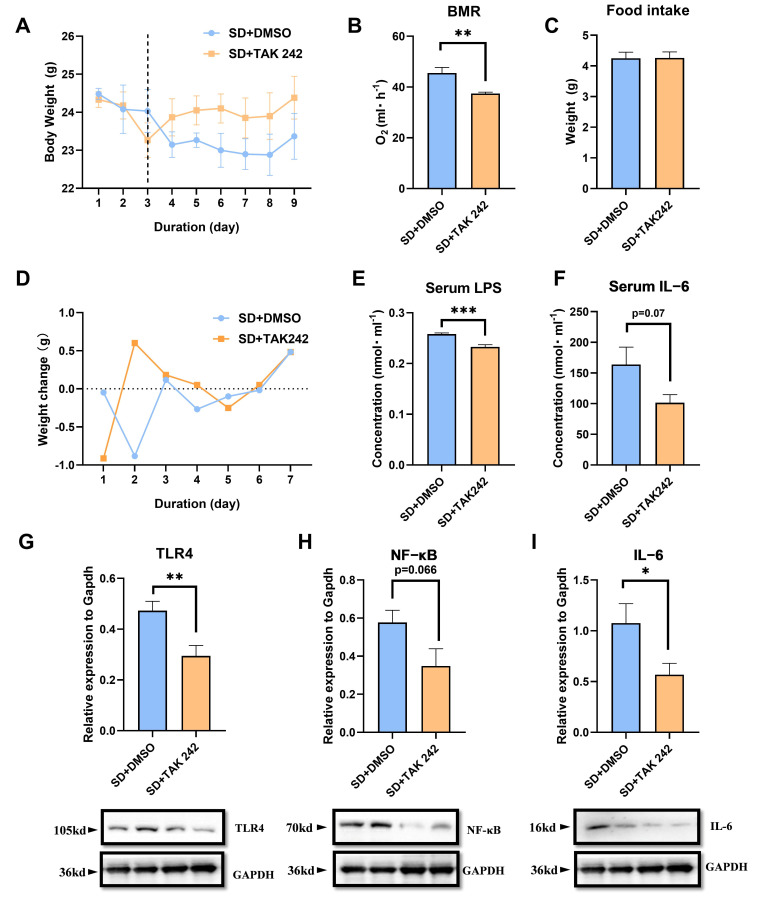
Effects of inhibitor injection on SD mice: (**A**) body weight, (**B**) BMR, (**C**) daily average food intake, (**D**) body weight changes, (**E**) serum LPS level, (**F**) serum IL-6 level, (**G**–**I**) the protein levels of TLR4, NF-κB and IL-6 of muscle. Values are means ± SEM. *, *p* < 0.05, **, *p* < 0.01, ***, *p* < 0.001.

The decrease in body weight caused by SD was significantly inhibited after the intraperitoneal injection of TAK 242 (*t*_10_ = 2.352, *p* = 0.0318, Figure 6A), but there was no significant difference in the daily food intake between the two groups of mice (*t*_10_ = 2.502, *p* > 0.05, Figure 6C). This effect may be attributed to the alleviation of the increase in the BMR of mice induced by SD after the injection of the inhibitor (*t*_10_ = 3.56, *p* = 0.0052, Figure 6B). Additionally, the increase in serum IL-6 (*t*_10_ = 1.988, *p* = 0.075, Figure 6E) and LPS (*t*_10_ = 5.438, *p* = 0.0003, Figure 6F) levels caused by SD was not observed after the injection of TAK 242. The results of protein levels revealed that the activation of the TLR4/NF-κB/IL-6 signaling pathway in skeletal muscle was inhibited (TLR4, *t*_10_ = 3.195, *p* = 0.009, Figure 6G; NF-κB, *t*_10_ = 2.06, *p* = 0.066, Figure 6H; IL-6, *t*_10_ = 2.301, *p* = 0.0442, Figure 6I).

## 4. Discussion

In this study, we examined the mechanisms by which SD affects energy metabolism, inflammation, and gut microbiota, as well as the potential protective role of exercise in mitigating these effects in mice. Results showed the following: (1) SD induces a surge in energy expenditure in mice via gut microbiota; (2) exercise enhances gut microbiota stability, mitigating the effects of SD; (3) exercise-induced butyrate elevation is key to improving gut stability and counteracting the adverse effects of SD. These findings suggest that exercise can counteract the energy expenditure surge induced by SD in mice by enhancing the stability of the gut microbiota.

1. SD Increased energy expenditure in Mice

Our results showed that mice in the SD group exhibited significantly higher energy expenditure but lower body weight than those in the CTR group. These findings are consistent with our previous work and can be attributed to the elevated energy expenditure that surpasses energy intake [56], indicating that SD enhances thermogenesis across multiple organs [16]. Existing studies have shown that the widely used multiple platform sleep deprivation (MMPM) method predominantly deprives mice of REM sleep and induces strong activation of the hypothalamic–pituitary–adrenal (HPA) axis, triggering stress responses that lead to a sharp increase in energy expenditure and subsequent body weight loss [57,58,59]. The rotating bar sleep deprivation system can also significantly reduce REM sleep in mice, thereby enhancing metabolism and leading to weight loss. And compared to the MMPM in a water environment, it imposes milder stress [60]. Although our SD method can partially activate the HPA axis and mediate some metabolic activities through it, existing studies suggest that it remains more suitable for investigating a range of physiological disturbances caused by chronic, mild stimuli, such as metabolic dysregulation and immune responses [25]. In addition, the inflammatory response triggered by SD has been identified as a key contributor to increased energy expenditure. This is likely due to the metabolic demands of immune activation and the thermogenic effects of pro-inflammatory cytokines, as previously reported [21,22,23]. Our measurements show elevated serum LPS level in SD mice, supporting this notion. Further analysis revealed that LPS activates the downstream TLR4/NF-kB/IL-6 signaling pathway in skeletal muscle, thereby triggering inflammation. Elevated concentrations of inflammatory factors, such as IL-6 and TNF-α, can significantly activate energy metabolism in adipose tissue, liver, and muscle, enhancing the oxidative metabolism of fat and glucose [61]. This increase in metabolic rate supports the energy demands of inflammatory and immune responses. Consistent with previous studies, SD-induced LPS further activated the inflammatory response in mice, elevating the levels of cellular inflammatory factors and altering the metabolic profiles of multiple organs [25,62].

2. Exercise alleviates harmful effects induced by SD

Exercise is widely recognized for its positive effects on sleep [37,38] and its ability to mitigate metabolic and cognitive impairments associated with SD [54,63]. However, direct evidence linking long-term exercise training to mitigation of SD-related adverse effects remains limited. Existing research suggest that exercise has beneficial effects after SD occurs [54,64], and it could be a potential therapeutic strategy to mitigate the metabolic disturbances caused by SD [65]. MICT has significant anti-inflammatory effects and metabolic regulatory functions. Combining MICT preconditioning with SD, our findings demonstrate that 21 days of MICT allows mice to effectively counteract the increased energy expenditure caused by SD. This protective effect is primarily attributed to the enhanced anti-inflammatory response conferred by prolonged exercise, which helps combat SD-induced inflammation, particularly inflammation associated with elevated LPS level [42]. Our results showed that the anti-inflammatory effects of exercise significantly reduced the SD-induced increase in LPS level, thereby preventing associated inflammatory responses. Additionally, our investigation demonstrated that the upregulation of thermogenesis induced by SD, is inhibited by exercise acclimation, as reported in our previous studies [16].

Previous studies have suggested that LPS suppression alleviates the detrimental effects of SD [25]. In our study, we used the TLR4 antagonist TAK 242, which effectively alleviated the increase in LPS levels induced by SD and inhibited the high-energy metabolism caused by inflammation while maintaining normal energy metabolism and preventing weight loss. These findings further determined the role of exercise in enhancing the body’s resistance to LPS, thereby mitigating the damage caused by SD.

3. The stability of the gut microbiota directly influences the body’s energy metabolism and immune function

Exercise has demonstrated a capacity to modulate gut microbiota, promoting the abundance of beneficial bacterial strains [66]. After MICT, we observed a substantial improvement in gut microbiota stability, counteracting the SD-induced loss of β-diversity. Specifically, exercise restored the reduced abundance of the *Lachnospiraceae_NK4A136_group* and *Lachnospiraceae-UCG-006* caused by SD. Previous studies have also shown that, despite using different SD protocols compared to ours, SD consistently led to a reduction in the abundance of *Lachnospiraceae* [67,68]. These are beneficial bacteria, primarily functioning to produce SCFAs [69]. In particular, *Lachnospiraceae-UCG-006* is capable of producing butyrate, which improves health by reducing pathogen colonization and toxicity. It plays an important role in maintaining gut health, regulating the immune system, and influencing host metabolism [70]. This suggests that changes in gut microbiota composition partially mediate the protective effects of exercise against the adverse impacts of SD.

LPS and SCFAs are key metabolites produced by gut microbiota and play a vital role in regulating the body’s inflammatory state [71,72]. Exercise has been shown to increase the abundance of SCFAs-producing bacteria, thereby elevating SCFA levels [46], which may counterbalance the effects of SD on SCFAs production [73]. Our targeted analysis of SCFAs revealed that exercise preconditioning inhibited the SD-induced reduction in butyric acid levels. Butyric acid, a key SCFA, is well known for its powerful anti-inflammatory effects [74]. Previous studies have indicated that butyrate supplementation can reduce LPS-induced inflammation by upregulating IL-10 and suppressing levels of pro-inflammatory cytokines, such as TNF-α, IL-6, and IL-1β [75]. In our experiment, butyrate supplementation partially alleviated the increase in LPS levels induced by sleep deprivation, accompanied by a moderate reduction in inflammatory markers, to some extent, reducing energy expenditure in mice. Although butyrate supplementation did not fully prevent weight loss or BMR increase associated with SD, we hypothesize that this may be due to the thermogenic effects of butyrate in various organs [76]. Notably, consistent with previous findings [77], our 16S rRNA sequencing results indicate that butyrate supplementation also reversed the decline in gut microbiota β-diversity caused by SD. Interestingly, similar to exercise, butyrate supplementation helped stabilize the abundance of *Lachnospiraceae-UCG-006*, supporting the maintenance of gut microbiota structure.

## 5. Conclusions

In summary, long-term exercise can effectively counteract the elevation of serum LPS induced by SD through modulation of gut microbiota, inhibition of inflammatory responses, and preservation of normal energy metabolism processes. Our results suggest that sustained exercise may serve as a viable intervention to mitigate the adverse effects associated with SD, providing robust support for strategies to prevent metabolic and inflammation-related diseases triggered by SD. Our findings provide new insights into the complex interactions between SD, energy expenditure, and gut microbiota. Unlike other studies that focus primarily on exploring the harmful effects of SD and alleviating its consequences post-occurrence, we propose exercise as a proactive intervention to counteract SD’s negative impacts and support metabolic and immune health. These results offer potential implications for the multiple benefits of exercise and suggest a non-pharmacological intervention strategy to mitigate health issues associated with SD caused by work or study demands.

## 6. Limitation

There are still several limitations in our study. Although the bar-based sleep deprivation system is relatively mild, it can still partially activate the HPA axis. However, our current study did not investigate the potential impact of this neuroendocrine response on energy metabolism. In future research, relevant indicators such as circulating corticosterone levels should be assessed to better understand the metabolic effects associated with bar-induced sleep deprivation. In addition, we only used a TLR4 inhibitor to verify the involvement of the LPS downstream signaling pathway in SD-induced inflammation. This provides limited mechanistic evidence, and future studies should aim to employ genetic knockout models to further elucidate the causal relationship between sleep deprivation, inflammation, and metabolic dysregulation. Although we identified certain signature microbial taxa through gut microbiota sequencing, their functional roles were not further explored. In subsequent studies, it is necessary to conduct mono-colonization experiments to validate the specific contributions of these key bacterial strains. Finally, this study was conducted in mice, which may not fully replicate the complexity of human physiology. Therefore, further clinical evidence will be essential to support the translational relevance and therapeutic implications of our findings in the context of sleep deprivation.

## Data Availability

The raw amplicon sequencing datasets obtained in this study were deposited in the NCBI and are available under accession numbers from SRR32653911 to SRR32653934 and SRR32810868 to SRR32810891 (https://www.ncbi.nlm.nih.gov/bioproject/PRJNA1234622; https://www.ncbi.nlm.nih.gov/bioproject/PRJNA1240313, accessed on 21 March 2025).

## Supplementary Materials

The following supporting information can be downloaded at https://www.mdpi.com/article/10.3390/biom15060862/s1, Table S1: HIIT training program of mice; Table S2: MICT training program of mice; Table S3: H&M training program of mice; Figure S1. The effects of three intensities of exercise on inflammation and metabolism; Figure S2. The thermogenic and inflammatory pathway indicators of iBAT; Figure S3. The crosstalk between muscle and inducible iBAT; Figure S4. Functional prediction of differential microbiota; Figure S5. Heatmap of top 50 different microbiota genera of 4 groups in Experiment 2; Figure S6. X- and Y-axis activity of mice was monitored during BMR measurement in Experiment 2.

## Abbreviations

SD: sleep deprivation; MICT: moderate-intensity continuous training; HIIT: high-intensity interval training; SCFA: short-chain fatty acids.
