# Supplementary material for: Long-Term Exercise Mitigates Energy Expenditure and Inflammatory Responses Induced by Sleep Deprivation in Mice
Source: Biomolecules. 2025 Jun 13;15(6):862. doi: 10.3390/biom15060862 (PMC12190197; doi:10.3390/biom15060862)
Supplement: Supplementary file 1 [file biomolecules-15-00862-s001.zip › biomolecules-3610642-supplementary-conversion.pdf]

Supplementary

Attached Table S1: HIIT training program of mice

HIIT										
Time	Warm-up 40~50% VO_{2max}		Circle I 85~90%VO_{2max}		Circle II 65~70%VO_{2max}		Cool-down 30~40% VO_{2max}		Distance (m)	Duration (min)
	Seep (m/min)	Time (min)	Speed (m/min)	Time (min)	Speed (m/min)	Time (min)	Speed (m/min)	Time (min)		
Week 1			22						770×5	47×5
Week 2	10	4	23	4×4	15	6×4	6	3	786×5	47×5
Week 3			24						802×5	47×5

Attached Table S2: MICT training program of mice

MICT								
Time	Warm-up 40~50% VO_{2max}		Train 65~70%VO_{2max}		Cool-down 30~40% VO_{2max}		Distance (m)	Duration (min)
	Seep (m/min)	Time (min)	Speed (m/min)	Time (min)	Speed (m/min)	Time (min)		
Week 1							808×5	
Week 2	10	4	15	50	6	3	808×5	57
Week 3							808×5	

Attached Table S3: H&M training program of mice

H&M										
Time	Warm-up 40~50% VO_{2max}		Circle I 85〜90% VO_{2max}		Circle II 65~70%VO_{2max}		Cool-down 30~40% VO_{2max}		Distance (m)	Duration (min)
	Speed (m/min)	Time (min)	Speed (m/min)	Time (min)	Speed (m/min)	Time (min)	Speed (m/min)	Time (min)		
Week 1 1, 3, 5 D			22	4×4	15	6×4			770×3	47×3
Week 1 2, 4 D			/	/	15	50			808×2	57×2
Week 2 1, 3, 5 D	10	4	23	4×4	15	6×4	6	3	786×3	47×3
Week 2 2, 4 D			/	/	15	50			808×2	57×2
Week 3 1, 3, 5 D			24	4×4	15	6×4			802×3	47×3
Week 3 2, 4 D			/	/	15	50			808×2	57×2

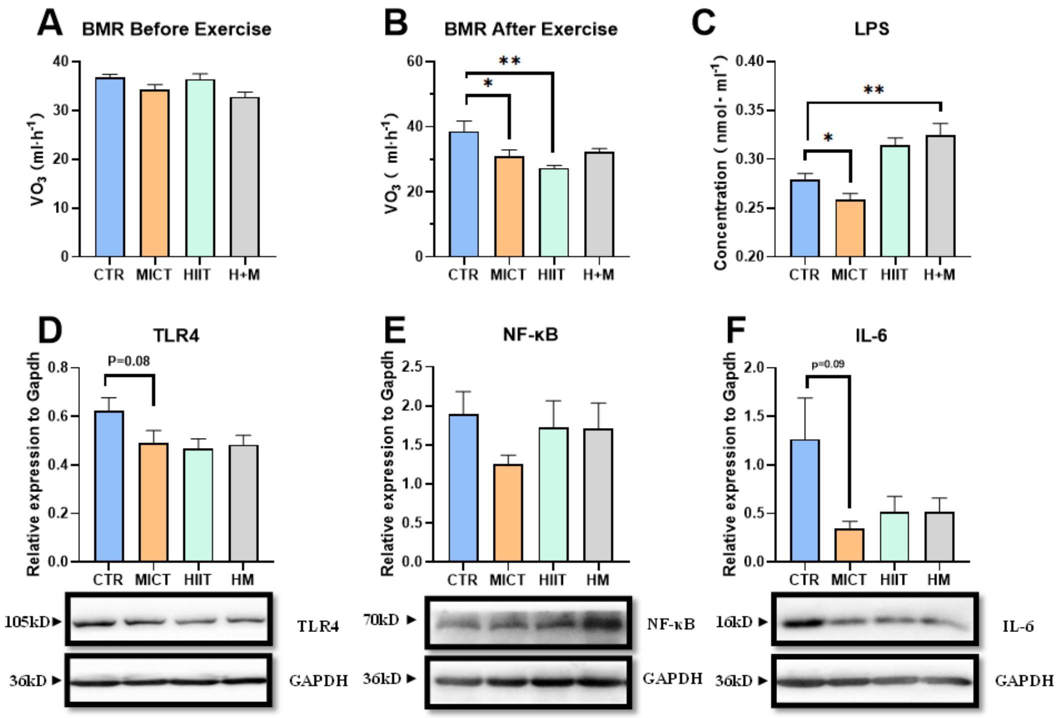

Figure S1. The effects of three intensities of exercise on inflammation and metabolism.: A-C BMR before exercise, BMR after exercise, serum LPS. D-F the protein levels of TLR4, NF-κB, and IL-6 of muscle. Values are means ± SEM. *, $p < 0.05$, **, $p < 0.01$.

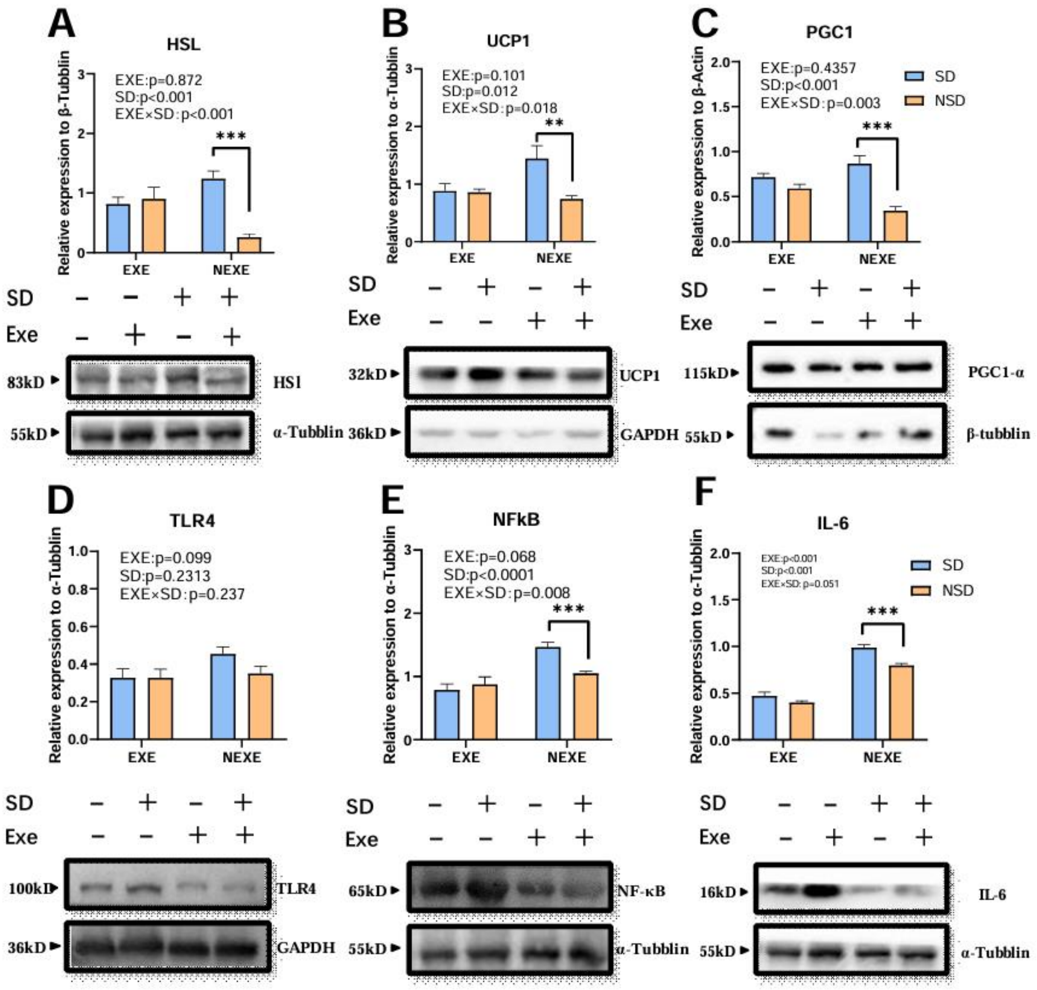

Figure S2. The thermogenic and inflammatory pathway indicators of iBAT.: A-F the protein levels of HSL, UCP1, PGC1, TLR4, NF-κB and IL-6 of iBAT. Values are means ± SEM. *, $p < 0.05$, **, $p < 0.01$.

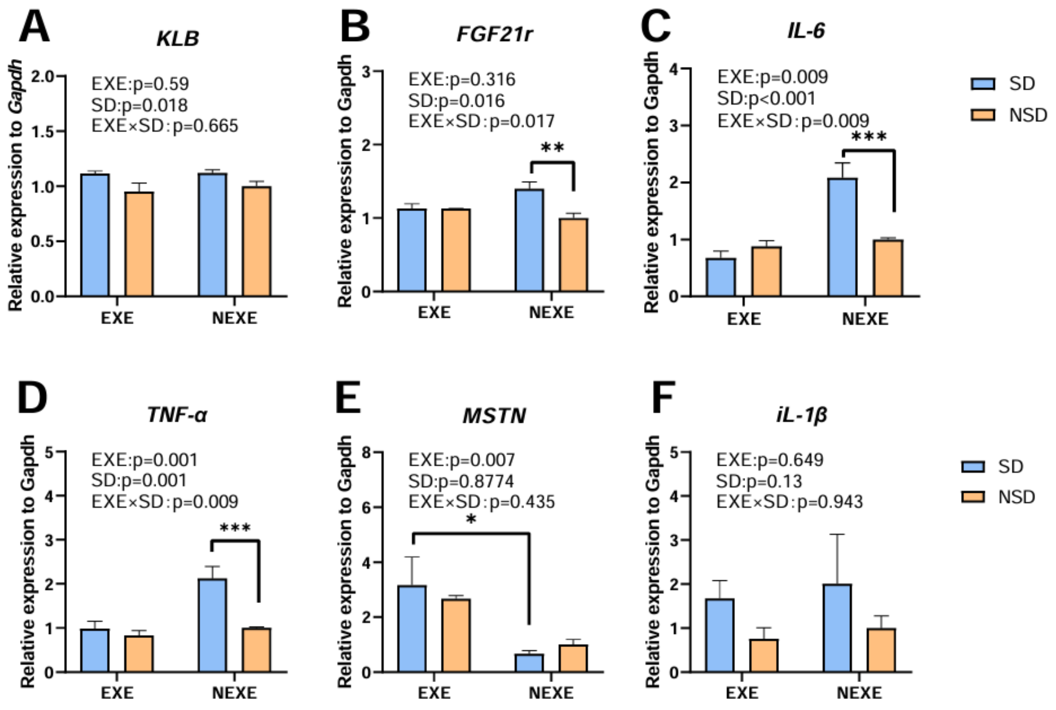

Figure S3. The crosstalk between muscle and inducible iBAT: A-D the *KLB*, *FGF21r*, *IL-6*, *TNF-α* gene expression levels of iBAT. E, F the *MSTN* and *IL-1β* gene expression levels of iBAT. Values are means ± SEM. *, $p < 0.05$, **, $p < 0.01$, ***, $p < 0.001$.

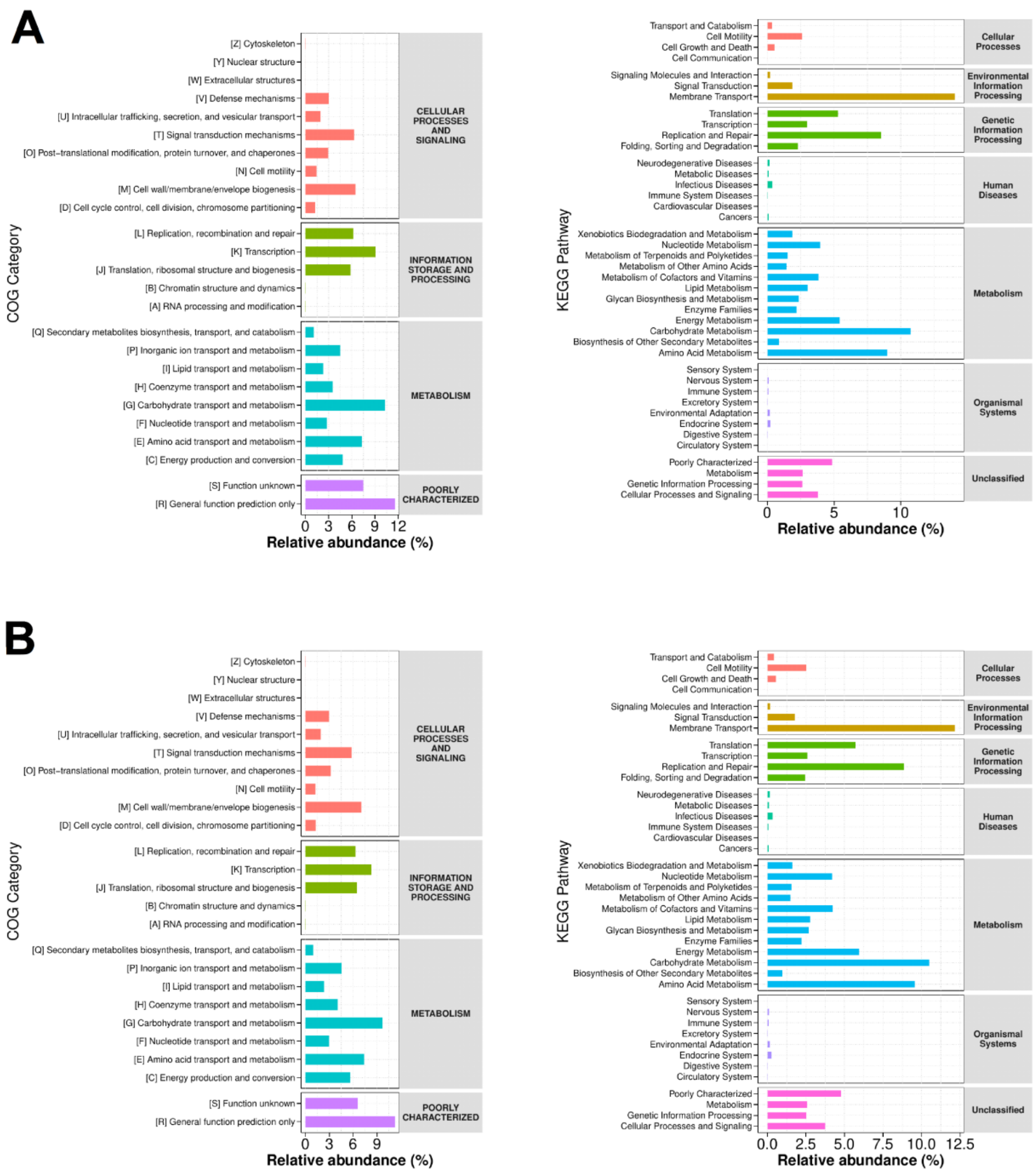

Figure S4. Functional prediction of differential microbiota: A-B KEEG and COG functional prediction of 2 experiment.

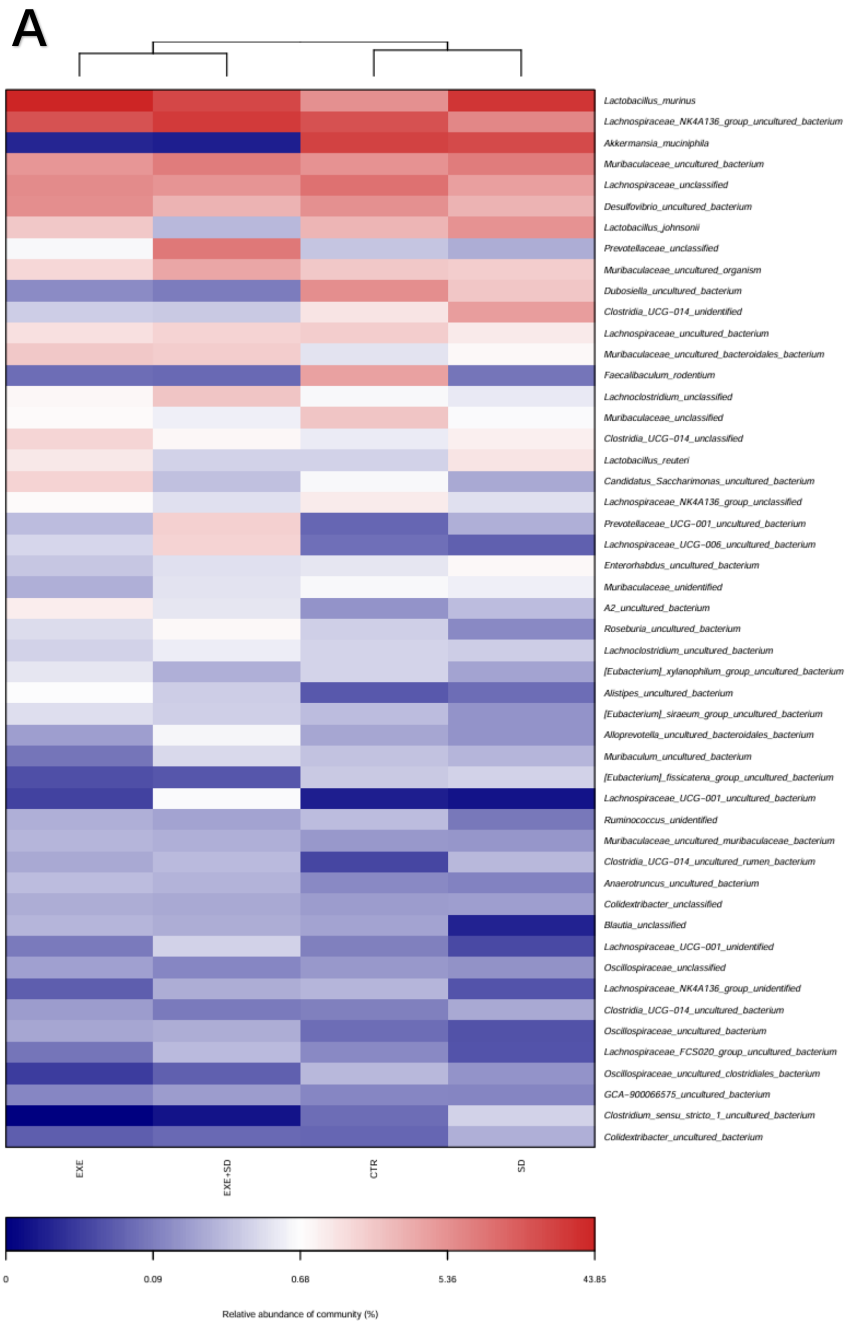

Figure S5. Heatmap of top 50 different microbiota genera of 4 groups in Experiment 2.

A

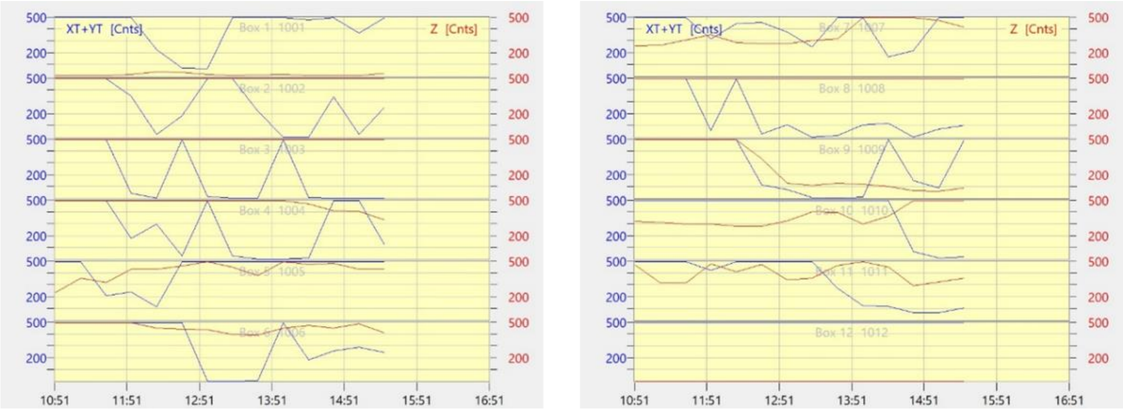

Figure S6. X- and Y-axis activity of mice was monitored during BMR measurement in Experiment 2.
