# Supplementary figures and images for: Long-Term Exercise Mitigates Energy Expenditure and Inflammatory Responses Induced by Sleep Deprivation in Mice
Source: Biomolecules. 2025 Jun 13;15(6):862. doi: 10.3390/biom15060862 (PMC12190197; doi:10.3390/biom15060862)

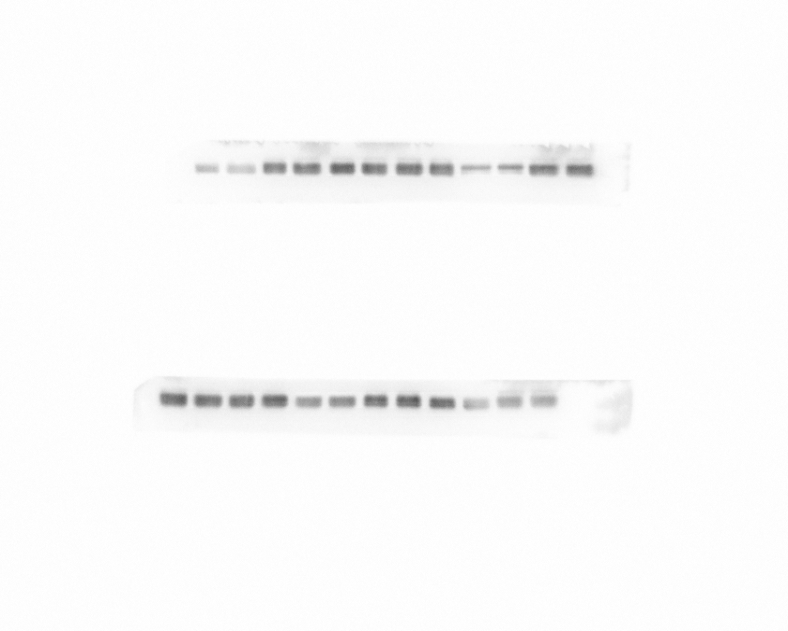

Supplement: Supplementary file 1 [file biomolecules-15-00862-s001.zip › biomolecules-3610642-original images/WB images/F1/Figure1 GAPDHú¿1ú⌐.bmp]

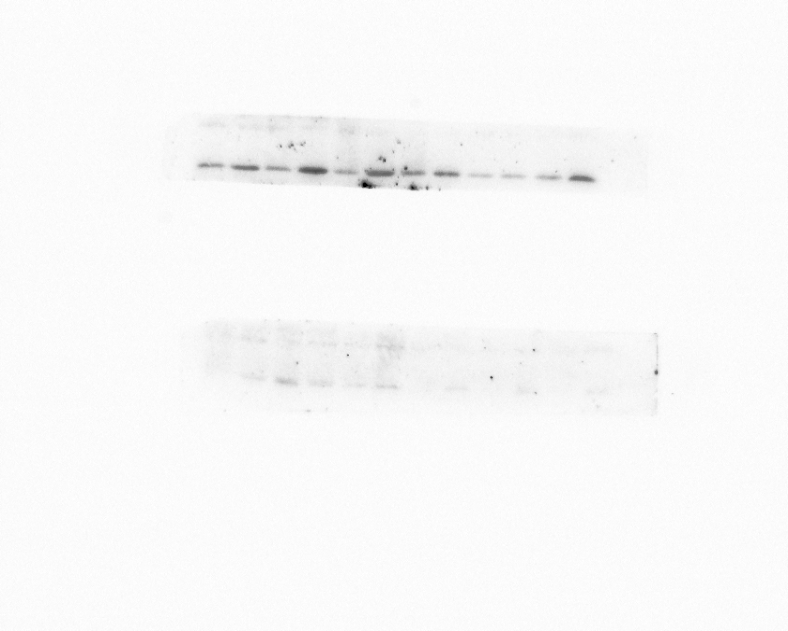

Supplement: Supplementary file 1 [file biomolecules-15-00862-s001.zip › biomolecules-3610642-original images/WB images/F1/Figure1 IL-6 ú¿1ú⌐.tif]

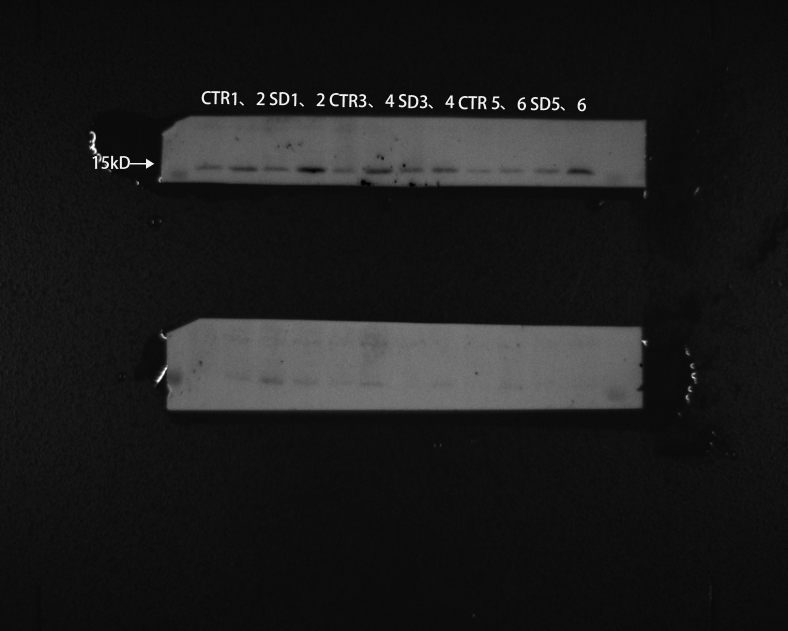

Supplement: Supplementary file 1 [file biomolecules-15-00862-s001.zip › biomolecules-3610642-original images/WB images/F1/Figure1 IL-6 ú¿2ú⌐.tif]

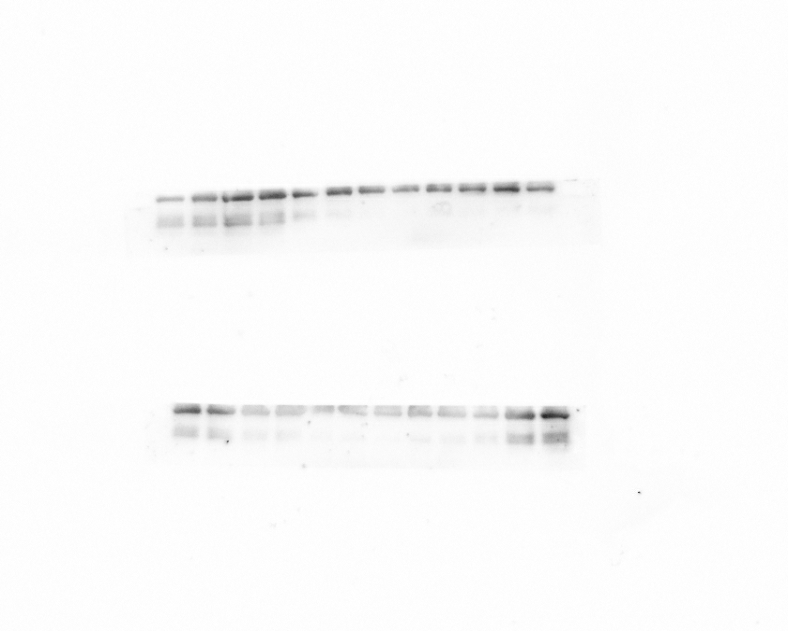

Supplement: Supplementary file 1 [file biomolecules-15-00862-s001.zip › biomolecules-3610642-original images/WB images/F1/Figure1 NF-kBú¿1ú⌐.bmp]

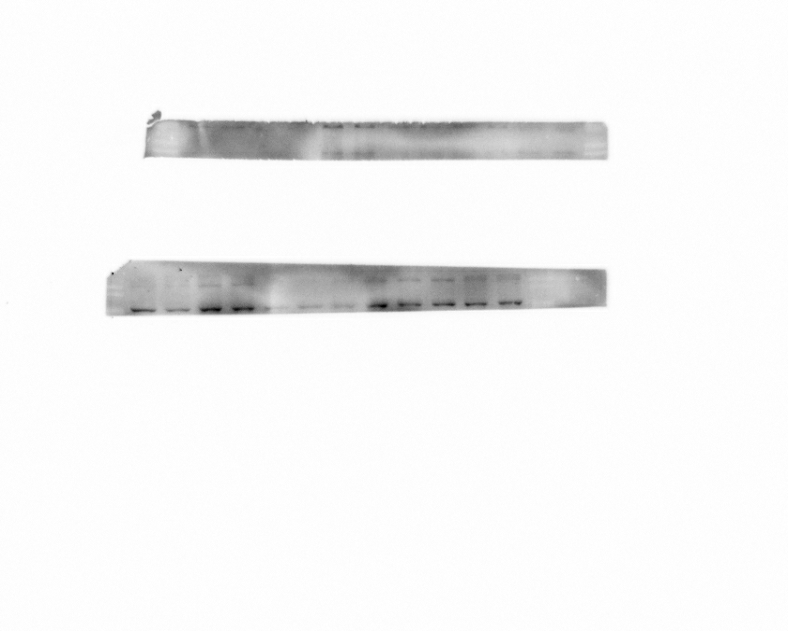

Supplement: Supplementary file 1 [file biomolecules-15-00862-s001.zip › biomolecules-3610642-original images/WB images/F1/Figure1 TLR4 ú¿1ú⌐.bmp]

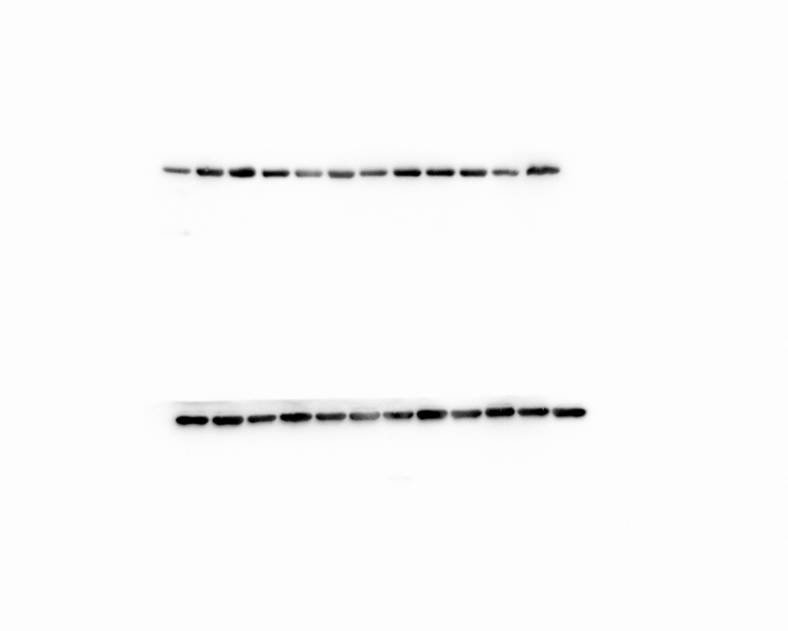

Supplement: Supplementary file 1 [file biomolecules-15-00862-s001.zip › biomolecules-3610642-original images/WB images/F2/Figure 2 GAPDH(1).tif]

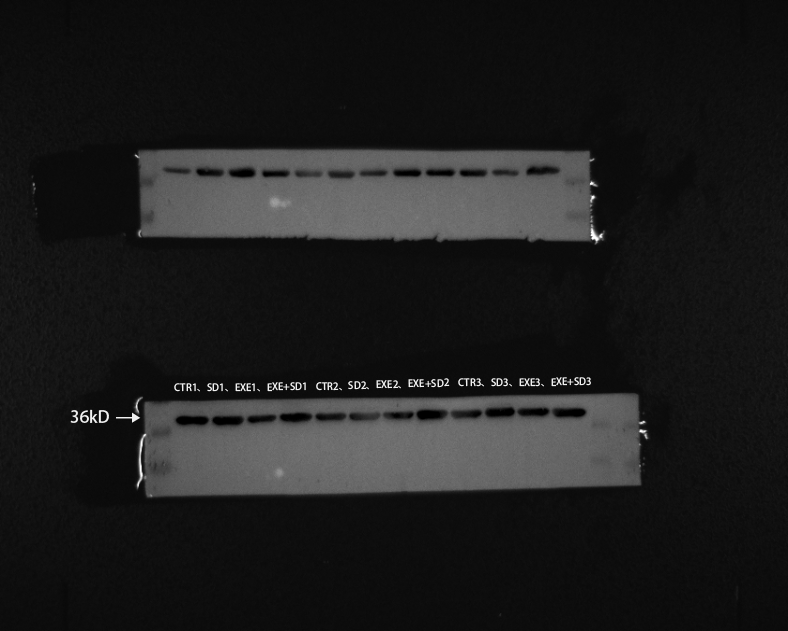

Supplement: Supplementary file 1 [file biomolecules-15-00862-s001.zip › biomolecules-3610642-original images/WB images/F2/Figure 2 GAPDH(2).tif]

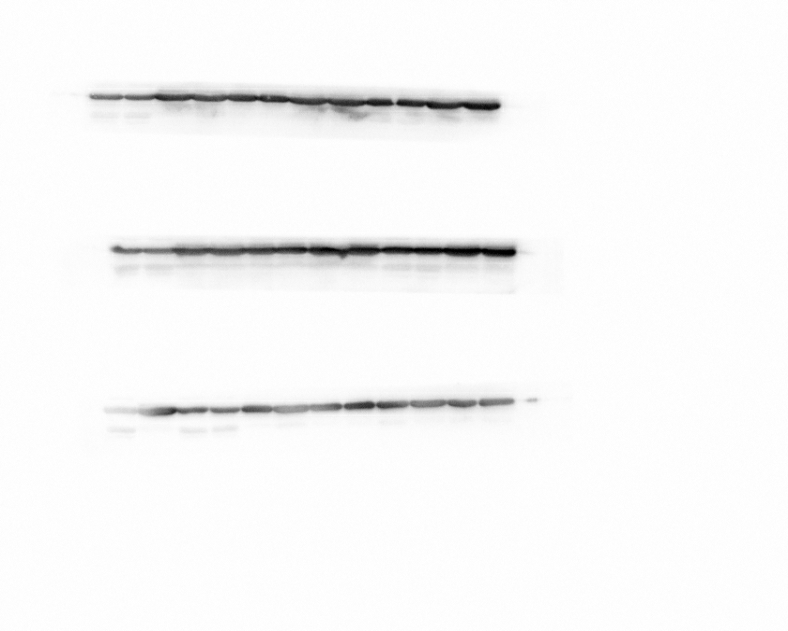

Supplement: Supplementary file 1 [file biomolecules-15-00862-s001.zip › biomolecules-3610642-original images/WB images/F2/Figure 2 GAPDH(3).bmp]

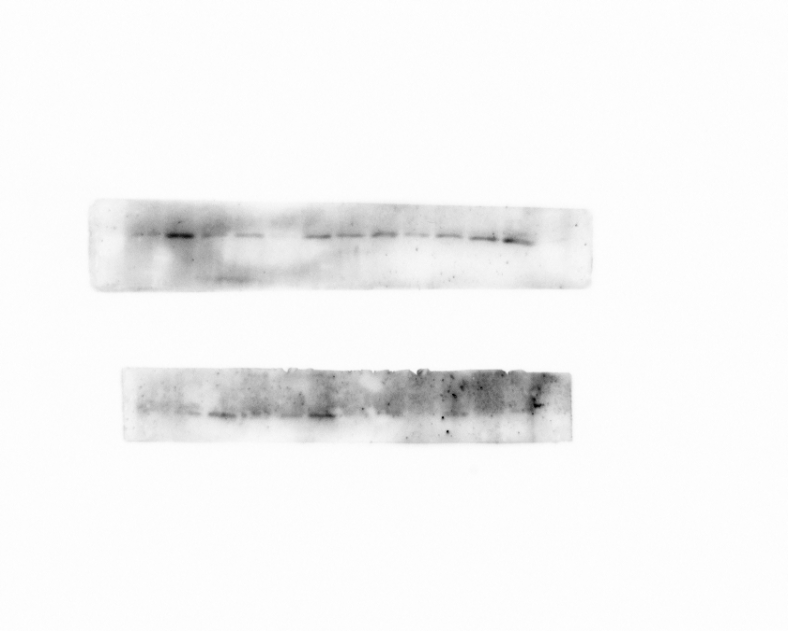

Supplement: Supplementary file 1 [file biomolecules-15-00862-s001.zip › biomolecules-3610642-original images/WB images/F2/Figure 2 IL-6 (1).tif]

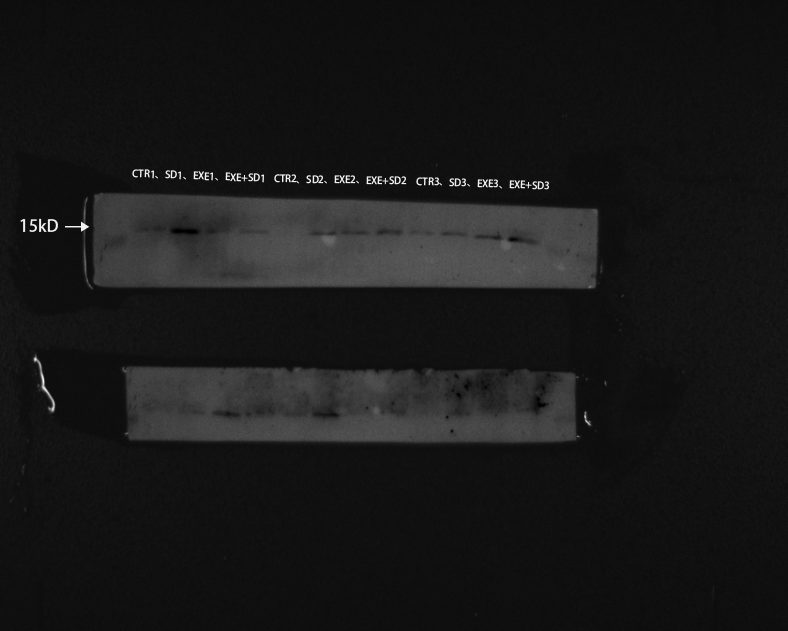

Supplement: Supplementary file 1 [file biomolecules-15-00862-s001.zip › biomolecules-3610642-original images/WB images/F2/Figure 2 IL-6 (2).tif]

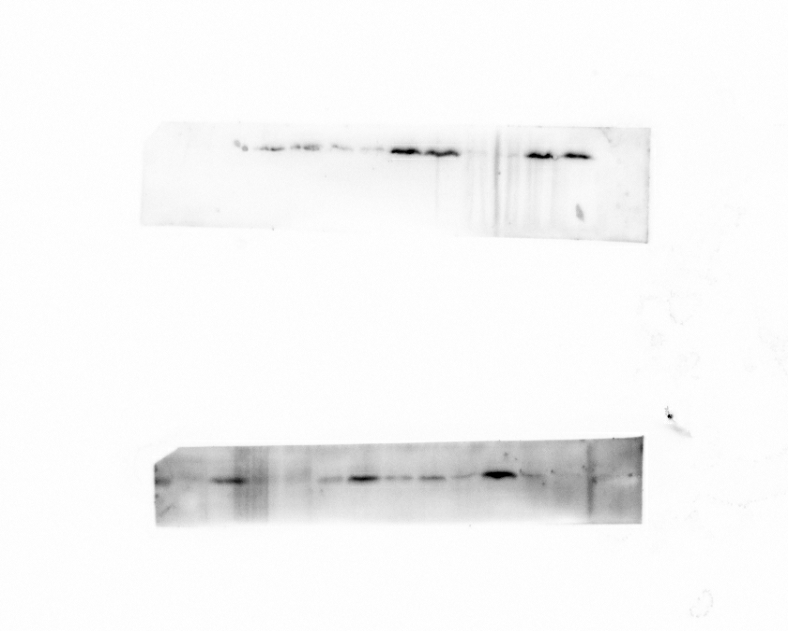

Supplement: Supplementary file 1 [file biomolecules-15-00862-s001.zip › biomolecules-3610642-original images/WB images/F2/Figure 2 IL-6 (3).bmp]

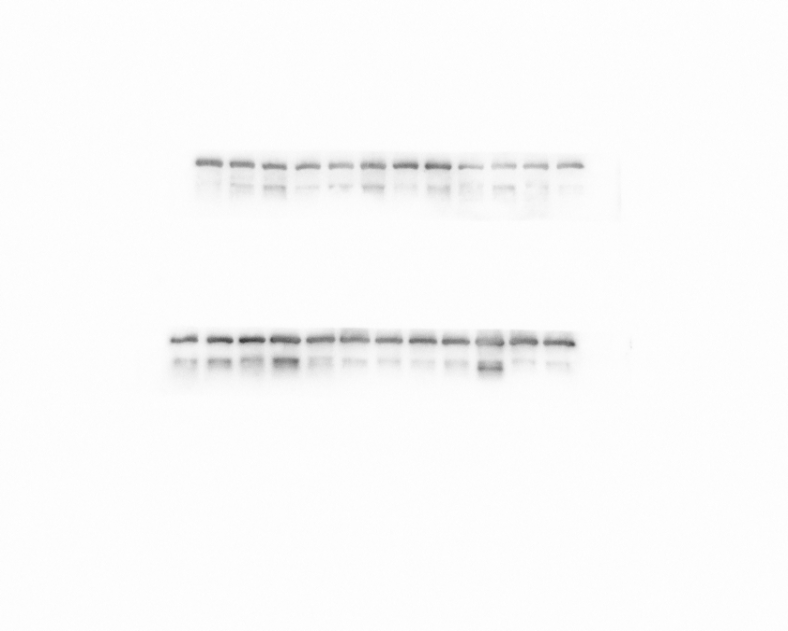

Supplement: Supplementary file 1 [file biomolecules-15-00862-s001.zip › biomolecules-3610642-original images/WB images/F2/Figure 2 NF-kB (1).tif]

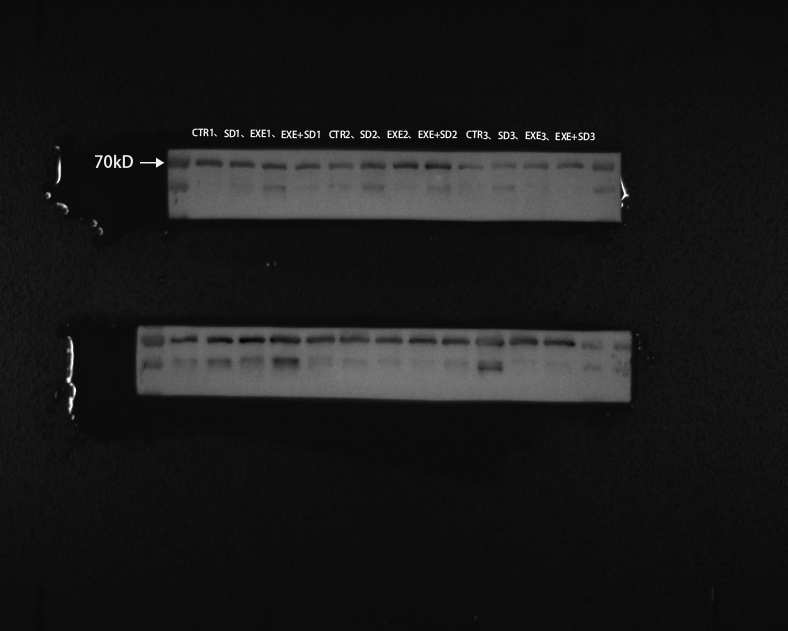

Supplement: Supplementary file 1 [file biomolecules-15-00862-s001.zip › biomolecules-3610642-original images/WB images/F2/Figure 2 NF-kB (2).tif]

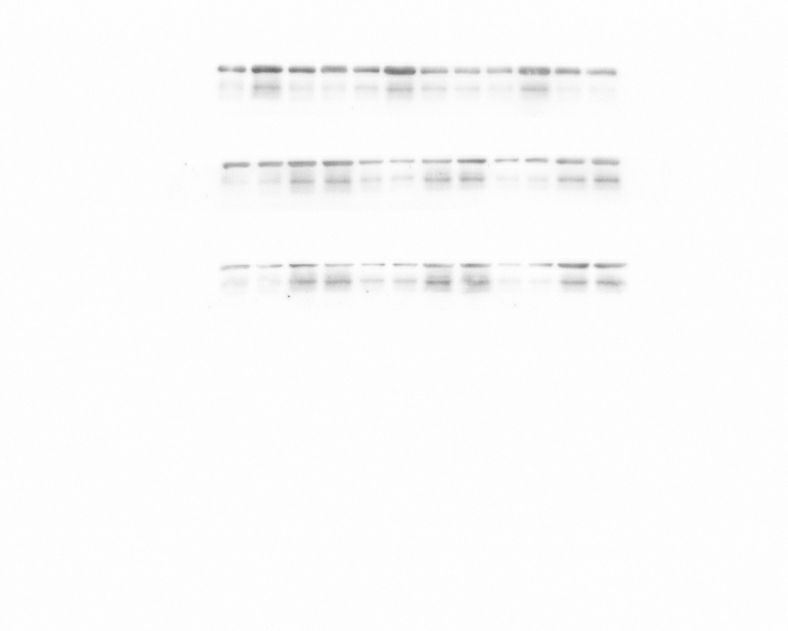

Supplement: Supplementary file 1 [file biomolecules-15-00862-s001.zip › biomolecules-3610642-original images/WB images/F2/Figure 2 NF-kB (3).bmp]

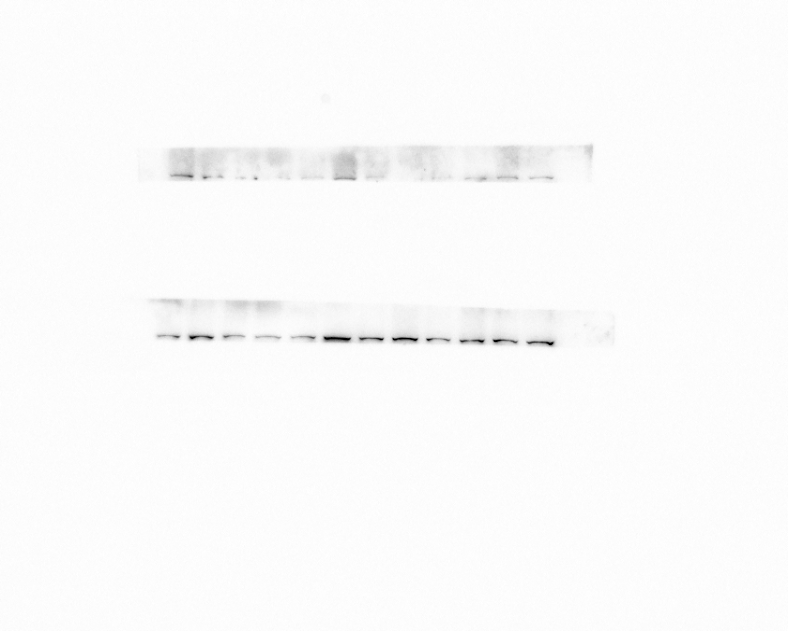

Supplement: Supplementary file 1 [file biomolecules-15-00862-s001.zip › biomolecules-3610642-original images/WB images/F2/Figure 2 TLR4 (1).tif]

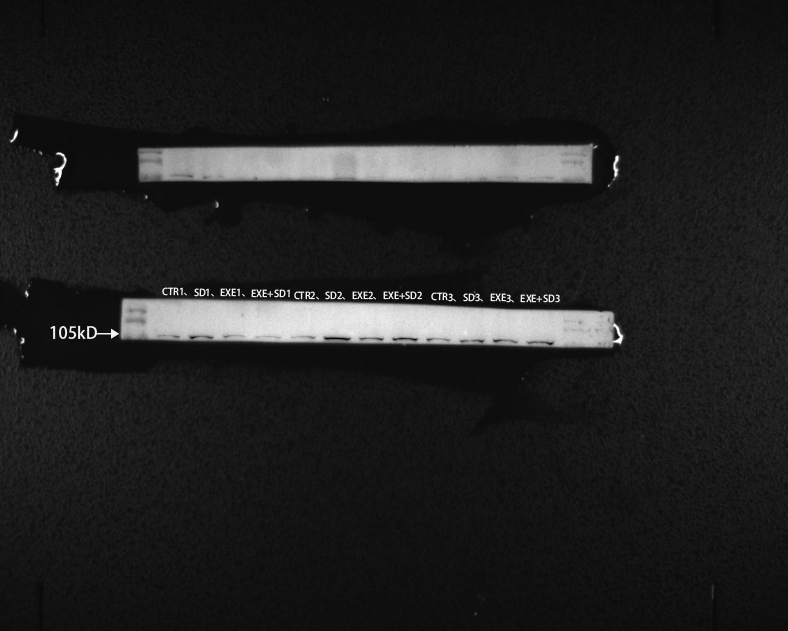

Supplement: Supplementary file 1 [file biomolecules-15-00862-s001.zip › biomolecules-3610642-original images/WB images/F2/Figure 2 TLR4 (2).tif]

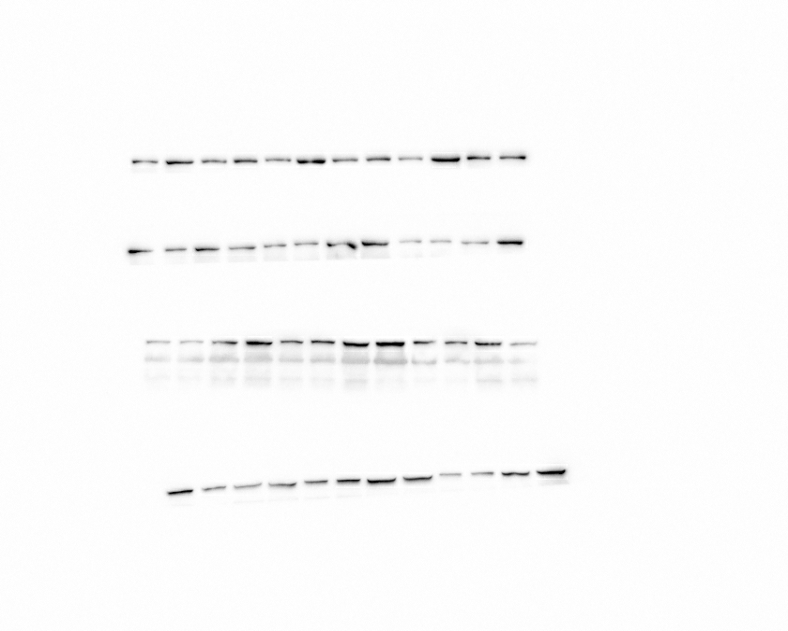

Supplement: Supplementary file 1 [file biomolecules-15-00862-s001.zip › biomolecules-3610642-original images/WB images/F2/Figure 2 TLR4 (3).bmp]

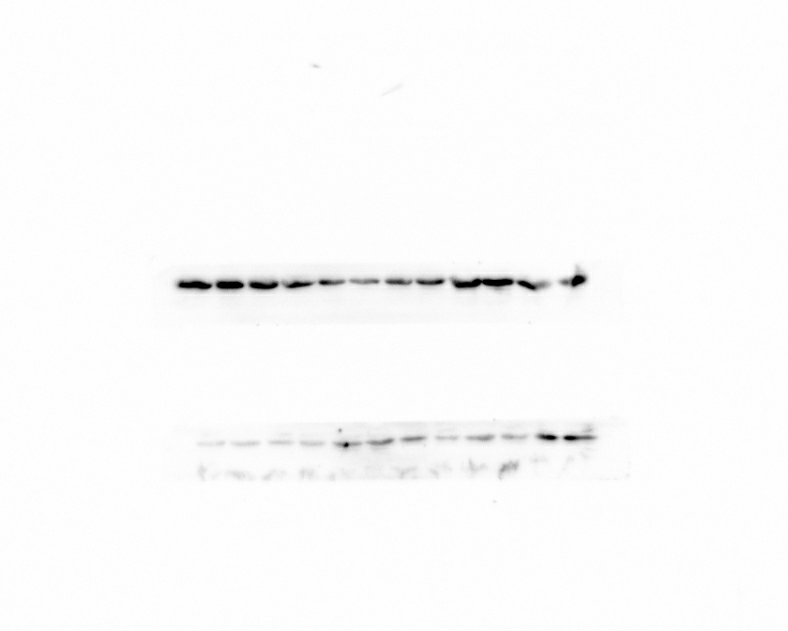

Supplement: Supplementary file 1 [file biomolecules-15-00862-s001.zip › biomolecules-3610642-original images/WB images/F4/Figure 4 GAPDHú¿1ú⌐.bmp]

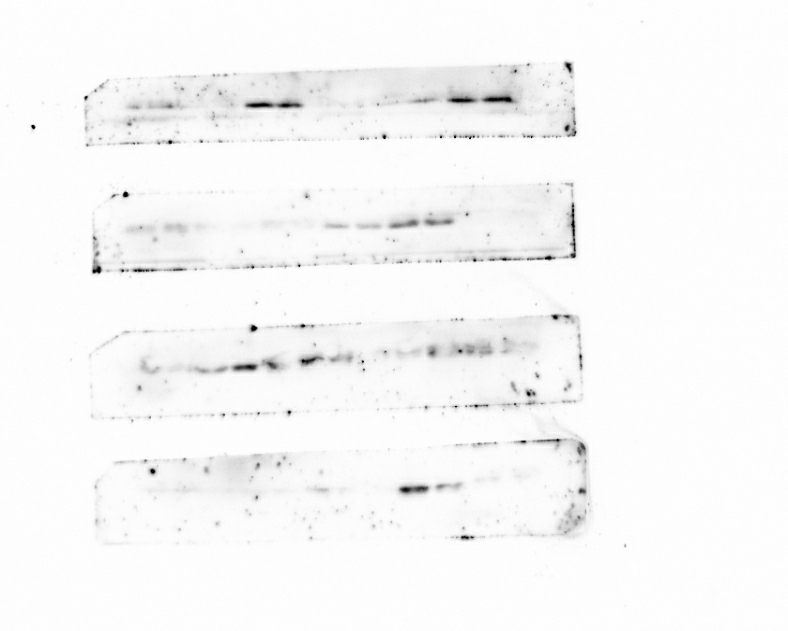

Supplement: Supplementary file 1 [file biomolecules-15-00862-s001.zip › biomolecules-3610642-original images/WB images/F4/Figure 4 IL-6 ú¿1ú⌐.bmp]

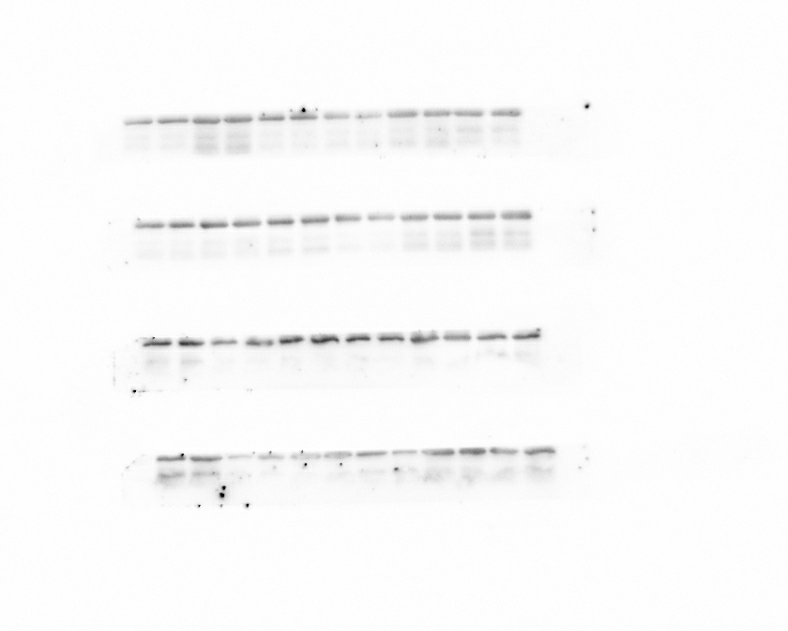

Supplement: Supplementary file 1 [file biomolecules-15-00862-s001.zip › biomolecules-3610642-original images/WB images/F4/Figure 4 NF-kBú¿1ú⌐.bmp]

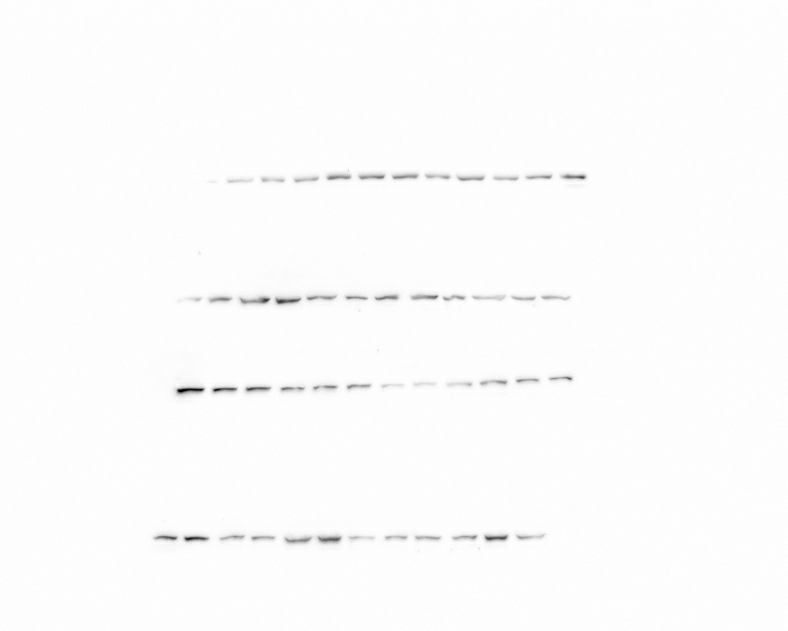

Supplement: Supplementary file 1 [file biomolecules-15-00862-s001.zip › biomolecules-3610642-original images/WB images/F4/Figure 4 TLR4 ú¿1ú⌐.bmp]

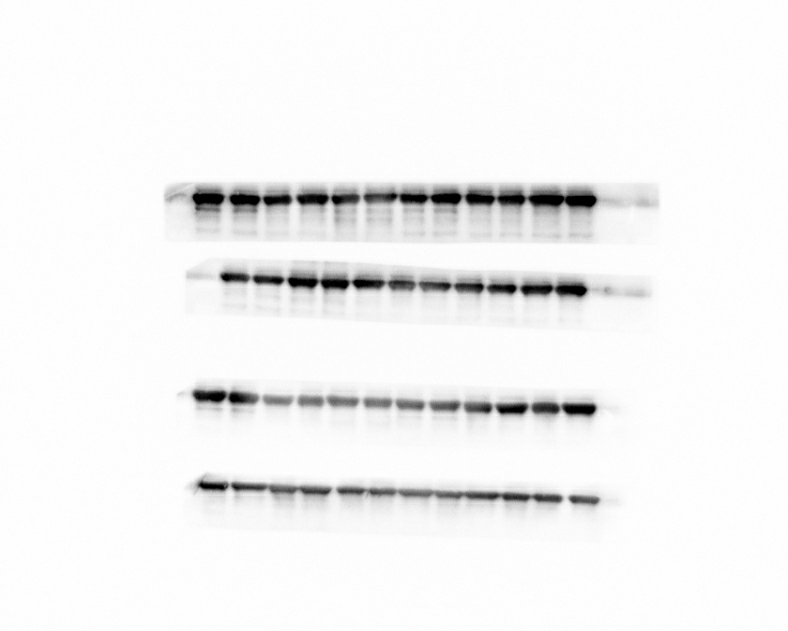

Supplement: Supplementary file 1 [file biomolecules-15-00862-s001.zip › biomolecules-3610642-original images/WB images/F6/Figure6 GAPDHú¿2ú⌐.bmp]

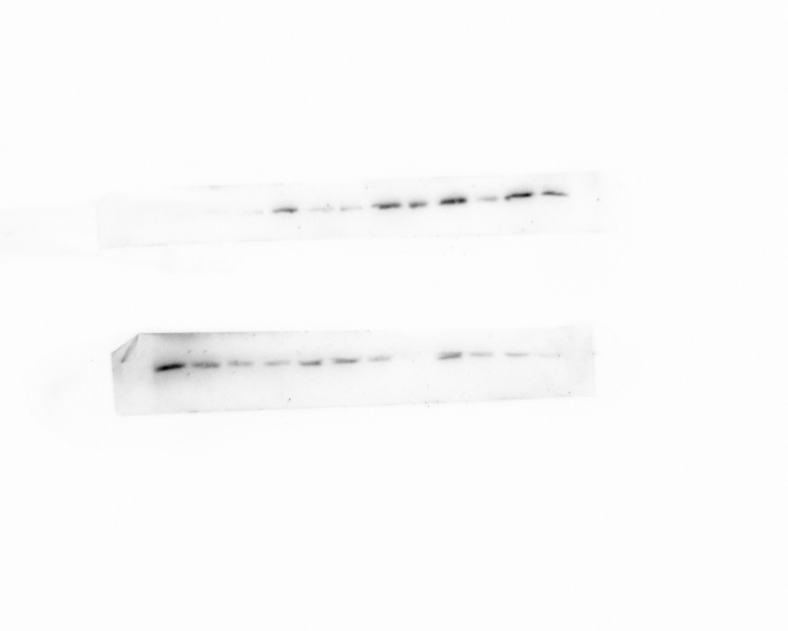

Supplement: Supplementary file 1 [file biomolecules-15-00862-s001.zip › biomolecules-3610642-original images/WB images/F6/Figure6 IL-6 ú¿1ú⌐.tif]

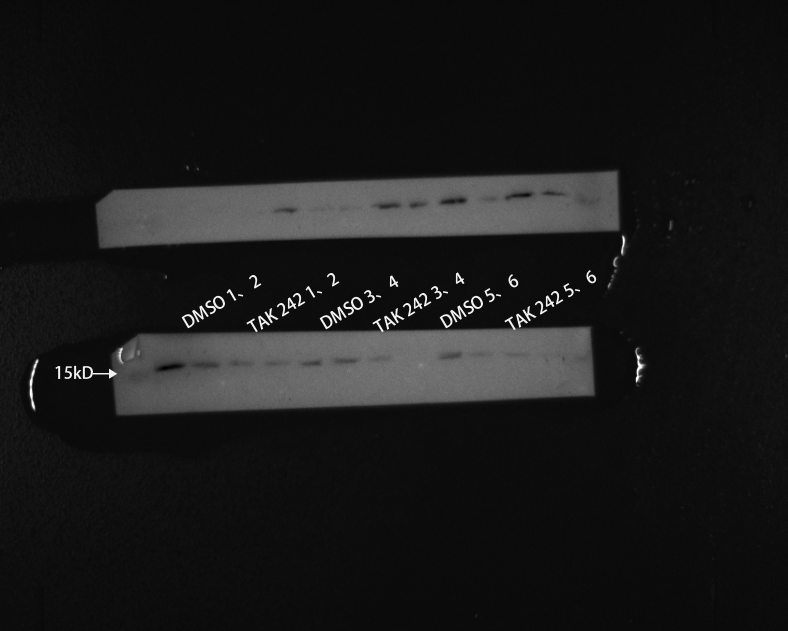

Supplement: Supplementary file 1 [file biomolecules-15-00862-s001.zip › biomolecules-3610642-original images/WB images/F6/Figure6 IL-6 ú¿2ú⌐.tif]

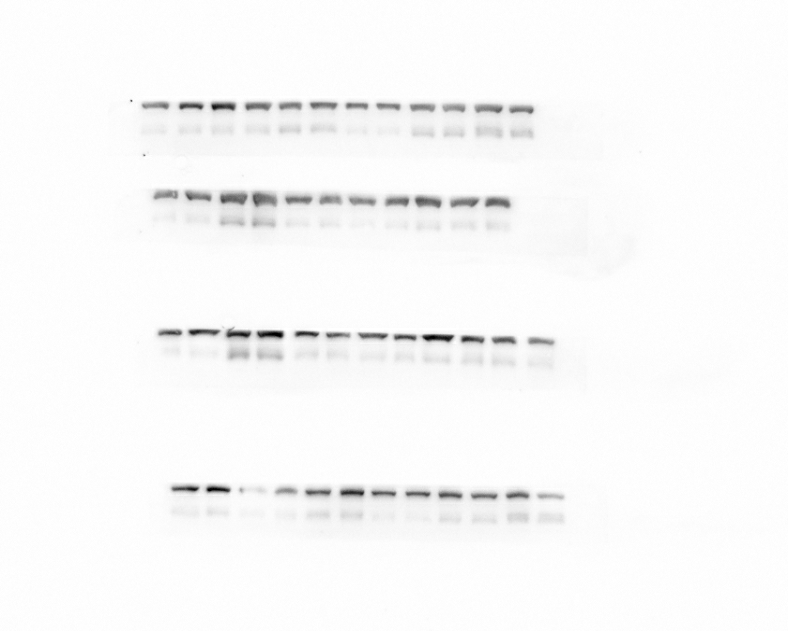

Supplement: Supplementary file 1 [file biomolecules-15-00862-s001.zip › biomolecules-3610642-original images/WB images/F6/Figure6 NF-kB(1).bmp]

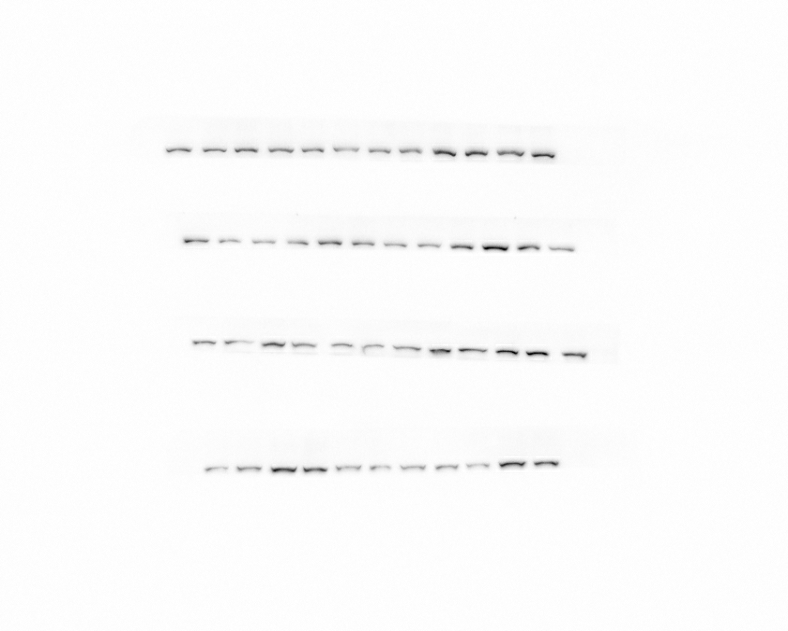

Supplement: Supplementary file 1 [file biomolecules-15-00862-s001.zip › biomolecules-3610642-original images/WB images/F6/Figure6 TLR4 ú¿1ú⌐.bmp]
